# Dendritic Pooling of Noisy Threshold Processes Can Explain Many Properties of a Collision-Sensitive Visual Neuron

**DOI:** 10.1371/journal.pcbi.1004479

**Published:** 2015-10-29

**Authors:** Matthias S. Keil

**Affiliations:** 1 Basic Psychology Department, University of Barcelona, Barcelona, Spain; 2 Institute of Brain, Bahaviour and Cognition (I3C), University of Barcelona, Barcelona, Spain; Université Paris Descartes, Centre National de la Recherche Scientifique, FRANCE

## Abstract

Power laws describe brain functions at many levels (from biophysics to psychophysics). It is therefore possible that they are generated by similar underlying mechanisms. Previously, the response properties of a collision-sensitive neuron were reproduced by a model which used a power law for scaling its inhibitory input. A common characteristic of such neurons is that they integrate information across a large part of the visual field. Here we present a biophysically plausible model of collision-sensitive neurons with *η*-like response properties, in which we assume that each information channel is noisy and has a response threshold. Then, an approximative power law is obtained as a result of pooling these channels. We show that with this mechanism one can successfully predict many response characteristics of the *Lobula Giant Movement Detector Neuron* (LGMD). Moreover, the results depend critically on noise in the inhibitory pathway, but they are fairly robust against noise in the excitatory pathway.

## Author Summary

Many different animals (from insects to primates) try to escape from collision threats, because it is very possible that the approaching object is a predator. The corresponding neurons in the various nervous systems must therefore detect such threats and signal when it is time to escape. Surprisingly, the neurons of different animals which selectively respond to approaching objects have very similar properties. It is therefore worthwhile to understand their underlying computational principles. A common characteristic of such neurons is that they receive (or integrate) information from the whole visual field. The integration process is carried out by the dendritic tree of the neuron. Here we present a computational model in which we assume that each of the input signals is contaminated by noise, as well as having a response threshold (which has to be crossed in order to evoke a response). Then, dendritic integration approximates a mathematical function (a power law) which is essential in our model for explaining the response characteristics of collision-sensitive neurons. Thus, noise is used in a constructive way for computing collision-sensitive responses. Power laws are furthermore found in many different contexts, and may consequently hint at the presence of noise and thresholds.

## Introduction

Noise is usually unwanted in signals, because it may introduce errors in transmitted messages. Yet, in the nervous system, noise is ubiquitous and ineluctable. In the visual system, for instance, photons arrive at the retina according to a Poisson process [1], giving rise to photon noise. The retinal photoreceptors absorb the photons by converting their energy into into a chemical signal. The chemical signal is then amplified and transduced into an electrical signal. The latter process introduces transduction noise [2]. Each subsequent stage of (visual) information processing adds further cellular, electrical, or synaptic noise [3, 4]. Nevertheless, the reliability at which organisms perform at the behavioural level evidences that the nervous system is designed in a way to handle noise well [5] while reducing power demand [6].

While commonly noise is just a nuisance, it can be used to solve computational problems. For example, a suitable amount of noise can help to push otherwise undetectable signals across the response threshold of a neuron (stochastic resonance [7]). In a similar way can noise smooth the threshold behaviour of a neuron. Assuming a constant noise level, an initially sub-threshold signal will elicit responses with an increasing probability if the signal amplitude approaches the firing threshold of the neuron [8, 9]. Threshold smoothing seems to be the crucial mechanism for transforming contrast-invariant, orientation-tuned input to cortical simple cells into contrast-invariant spike tuning. If spike traces are averaged across trials, then the threshold-linear relation will be effectively smoothed [10], approximating a power law around the threshold value [11, 12], and a linear relationship far from threshold. Threshold smoothing has thus been studied in the context of trial-averaging and in the average response of a neuronal population [8]. Here we propose that threshold smoothing may also occur at the single neuron level, for example when noisy and thresholded (pre-synaptic) inputs are integrated by the dendritic tree of a neuron. In this way, the synaptic input will be re-scaled according to an approximate power law, and we will use this property in a computational model that is aimed at explaining many characteristics of the *Lobula Giant Movement Detector* (LGMD) neuron of the locust.

The LGMD is a well-studied visual neuron that responds best to objects which approach one of the locust’s compound eyes on a direct collision course [13–15]. During an approach with constant speed, the response curve of the LGMD gradually increases to a maximum, and ceases abruptly afterwards [16, 17]. The response maximum (at time *t*_max_) is usually reached before the projected *time of collision* (“ttc” or *t*_*c*_). Yet it may also occur at or even after *t*_*c*_, especially if the approaching object is very fast or very small [18, 19]. The activity peak can furthermore be related to the timing of the locust’s escape jump [20].

The prevailing model for describing LGMD responses is the so-called Eta-function (“*η*”) ([16]; but see [21]). For simplicity, assume that a circular object (with diameter 2*l*) approaches the locust eye with a constant velocity *v*. Then, *η* is defined by multiplying two time-dependent functions. The first one, *exp*(−*α*Θ), depends on the *angular size* Θ(*t*) that the object projects on the eye at time *t*, and acts inhibitory (*α* is a positive constant). The second function provides excitatory input to the LGMD, and is denoted by $d\Theta /dt=\dot{\Theta }$. It represents the *angular velocity* or *rate of expansion*, respectively. So, taken together,

$$\begin{array}{c} \eta \left(\underset{\equiv T=T(t)}{\underset{︸}{t+\delta }}\right)=\dot{\Theta }\left(T\right)\cdot \text{exp}\left[-\alpha \Theta \left(T\right)\right]\; \end{array}$$ (1)

where *δ* accounts for the neuronal delay between stimulation of the eye and the response of the LGMD. (If not otherwise indicated, *δ* = 0 is assumed for all subsequent simulations). The *η*-function makes two important predictions, which are experimentally supported (ref. [17], Methods Section): *(i)* The time *T*_rel_ = *t*_rel_ − *δ* between the peak at *T*_max_ = *t*_max_ + *δ* and collision (at *t*_*c*_) depends linearly on the *halfsize-to-velocity ratio* *l*/*v*. Because a maximum at *T*_max_ implies $\dot{\eta }=0$ we have

$$\begin{array}{c} \underset{T_{\text{rel}}}{\underset{︸}{t_{c}-T_{\text{max}}}}=\frac{l}{v}\alpha \end{array}$$ (2)

where the slope *α* > 0 and the intercept *δ* ≤ 0 could be experimentally determined (typical values: *α* = 4.7, *δ* = −27ms [17]). *(ii)* For *δ* = 0 the maximum of *η* would coincide with angular size Θ(*t*_max_) = 2arctan(1/*α*). The maximum LGMD activity *in vivo*, however, occurs at Θ(*T*_max_), that is *δ* milliseconds after the stimulus has reached Θ(*t*_max_).

How is *η* computed at the biophysical level? One hypothesis holds that the LGMD uses a logarithmic transformation (i.e. log*η*) in its dendritic tree [22], followed by exponentiation (i.e. exp[log*η*]) by means of active membrane conductances in its axon [23]. Accordingly, direct multiplication is bypassed by subtracting the logarithmically encoded signals $\text{log}\dot{\Theta }-\alpha \Theta$. Experimental results suggest nevertheless that decoding (of the logarithmically encoded membrane potential) is “approximated” by a third-order power law [22–24], rather than being an exact exponentiation. Below we see that a power law alone does not suffice, as *η* would predict that the LGMD’s response curves to approaching objects would be significantly distorted.

But then, which biophysical principles underlie the computations carried out by the LGMD? A possible answer comes in the guise of a simple model (“noisy *ψ*” or “*n-* *ψ*”) that is based on an equation which describes the membrane potential of the LGMD neuron. Like *η*, *n-* *ψ* also receives Θ(*t*) and $\dot{\Theta }(t)$ as input signals. The main difference, however, lies in their respectively proposed biophysical mechanisms: Whereas *η* relies on logarithmic encoding and decoding for implementing multiplication, *n-* *ψ* relies on the presence of noisy inhibitory channels with threshold mechanisms, and the nonlinear properties of the membrane potential equation Eq (9). In this way, *n-* *ψ* emphasizes the importance of signal integration (e.g., across the LGMD’s dendritic tree) in order to account for many reported experimental observations

## Methods and Models

### Angular Variables

For simplicity, we assume that a circular object with diameter 2*l* approaches an observer with a constant velocity *v*. If the object starts its approach at a distance *x*_0_ then the *time to collision* (ttc) is at *t*_*c*_ = *x*_0_/*v* (here we do not distinguish between *time to contact* and *time to collision*). At the time *t* < *t*_*c*_, the object spans an angular size Θ(*t*) on the retina of the observer,

$$\begin{array}{c} \Theta \left(t\right)=2\arctan\frac{l}{x(t)} \end{array}$$ (3)

where *x*(*t*) = *x*_0_ − *vt*. The angular velocity or *rate of expansion* is defined as the first temporal derivative,

$$\begin{array}{c} \dot{\Theta }\left(t\right)=\frac{2lv}{x^{2}\left(t\right)+l^{2}} \end{array}$$ (4)

### Maximum of the *η*-function

The time *T*_max_ = *t*_max_ + *δ* of the maximum of the *η*-function $\eta \equiv \dot{\Theta }\cdot \text{exp}(-\alpha \Theta )$ is obtained through $\dot{\eta }=(d\eta /dT)\cdot (dT(t)/dt)=(d\eta /dT)=0$. This is equivalent to $\overset{..}{\Theta }=\alpha {\dot{\Theta }}^{2}$ and yields Eq (2). Thus, the *relative* time of the maximum *T*_rel_ ≡ *t*_*c*_ − *T*_max_ depends linearly on the halfsize-to-velocity ratio *l*/*v* with slope *α* and intercept *δ*. Together with Eq (3), Eq (2) also implies that the response maximum always occurs at a fixed angular size,

$$\begin{array}{c} \Theta_{max}\equiv \Theta \left(T_{\text{max}}\right)=2\arctan\frac{l}{v(t_{c}-T_{\text{max}})}=2\arctan\frac{1}{\alpha } \end{array}$$ (5)

which only depends on *α*, but not on time.

### Simulation details

The Matlab environment was used for all simulations. The default parameters of the *n-* *ψ*-model were as follows: *β* = 1, *V*_*inh*_ = −0.005, *V*_*rest*_ = 10^−5^, *V*_*exc*_ = 1, *γ* = 500, *σ* = 0.25, Δ_0_ = 0.9, *ζ*_0_ = *ζ*_1_ = 0.95, *N* = 500 (nearly identical *n-* *ψ*-predictions are obtained already for *N* ⪆ 10). If different values from the latter were used, they would be indicated with each figure. Since *V*_*inh*_ ≈ *V*_*rest*_, inhibitory input acted by increasing the effective leakage conductance *β* (silent or shunting inhibition). In other words, inhibitory input will not show unless excitatory input is present at the same time. The seed of the random number generator was chosen identical for each simulation run. This means that the same sequence of random numbers was generated. Eq (10) was integrated with the 4th-order Runge-Kutta (“RK”) method using the step size 500*μs*. The stimulation time scale was set to Δ*t*_*stim*_ = 1*ms*. Or, expressed differently, angular size Θ Eq (3) and angular velocity $\dot{\Theta }$ Eq (4) were discretized with Δ*t*_*stim*_ (cf. Eqs (11) and (12). As a consequence, the membrane potential *V*(*t*) is far from its steady state *V*_∞_ Eq (10) at each time *t*. For this reason, and in order to maintain the consistency with its predecessor model (= *ψ*, see ref. [19]), *n*_*relax*_ = 250 relaxation time steps were intercalated at each *t*. A relaxation time step is just an RK step with frozen values of *g*_*exc*_ and *g*_*inh*_. *V*(*t*) can be driven in this way closer to its equilibrium solution. The influence of *n*_*relax*_ on the predictions of *n-* *ψ* is broken down in Section C in S1 Text.

The default parameters for the object approach were diameter 2*l* = 0.12*m*, approach velocity *v* = 6*m*/*s*, initial distance *x*_0_ = 0.3*m*, continuous stimulation (versus discrete stimulation, see [19]). Notice that Θ and $\dot{\Theta }$ were not computed from video sequences, but directly from Eqs (3) and (4), respectively.

## Results

### Can a power law undo logarithmic encoding?

Logarithmic encoding of Eq (1) means $\text{log}\dot{\Theta }-\alpha \Theta$ [16, 23]. By doing so, the LGMD could basically multiply: log(*x* ⋅ *y*) = *log*(*x*) + *log*(*y*). The result of multiplication is obtained by applying exp(⋅). Accordingly, the LGMD’s membrane potential should be exponentially scaled, but figure 4d in reference [23] rather suggests a power law. At least mathematically it is clear that (log*η*)^*p*^ is a rather poor approximation of exp(log*η*). But how would the predictions from *η* look like when this approximation was used in the context of approaching objects? In order to answer that, we “undo” logarithmic encoding with a power law of order *p* (let *g*_*p*_(*t*) = predicted LGMD response):

$$\begin{array}{c} g_{p}\left(t\right)={\left[\log\dot{\Theta }\left(t\right)-\alpha \Theta \left(t\right)\right]}^{p} \end{array}$$ (6)

The extremum of this function is either a maximum (for power law exponents *p* = 1, 3, 5, …) or a minimum (*p* = 2, 4, 6, …) at ${\dot{g}}_{p}=0$, that is

$$\begin{array}{c} p{\left[\log\dot{\Theta }-\alpha \Theta \right]}^{p-1}\left(\frac{\ddot{\Theta }}{\dot{\Theta }}-\alpha \dot{\Theta }\right)=0 \end{array}$$ (7)

Because of the factor $\left(\overset{..}{\Theta }/\dot{\Theta }-\alpha \dot{\Theta }\right)$ the extrema of *g*_*p*_ will still occur at the same time points as those of the *η*-function, which is good news. The bad news, however, is that the extrema will be maxima only for odd values of *p*, where *g*_*p*_(*t*) ≤ 0 for all *t*. Thus, if we wanted *g*_1, 3, 5, …_ to be positive, we would need to add an offset *o* to Eq (6) (say *a* such that *g*_*p*_(*t*) + *o* ≥ 0). For even values of *p*, *g*_*p*_ has minima with *g*_*p*_(*t*) ≥ 0 for all *t*. In the latter case, an operation such as *o* − *g*_*p*_(*t*) (with *o* ≥ max_*τ*_[*g*_*p*_(*τ*)], *τ* ≤ *t*_*c*_) would be needed to transform the minima into maxima.

**Fig 1:**
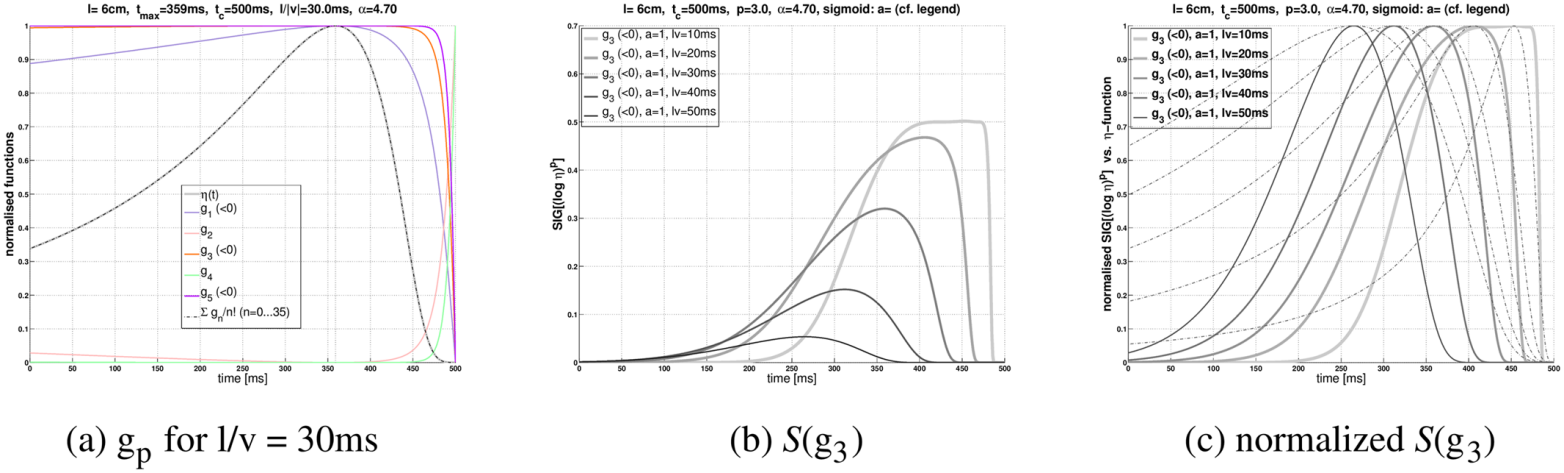
Logarithmic encoding of *η*(*t*). (**a**)The logarithmically encoded *η*-function $\text{log}\dot{\Theta }-\alpha \Theta$ is “approximately exponentiated” by a power law with exponent *p* according to *g*_*p*_(*t*) = [log*η*(*t*)]^*p*^ Eq (6). The corresponding curves of *g*_*p*_ (with *p* = 1,2,3,4,5 for *l*/*v* = 30ms) are denoted in the legend. For control, the sum $\sum_{p=1}^{35}g_{p}/n!$ (exponential series: dash-dotted curve), and the *η*-function without log-encoding (gray curve) are also shown. Note that the curves become flatter with increasing *p* (and also with increasing *l*/*v*—not shown), what could make the detection of the maxima more uncertain in the presence of noise (cf. Fig 2b). (**b**)Applying a sigmoidal function 𝒮(*x*) = [1 + exp(− *ax*)]^−1^ (with *a* = 1) to the properly normalized curves *g*_*p*_(*t*) (with *p* = 3) produces curves which resemble true LGMD responses Eq (8). The curves shown here correspond to different halfsize-to-velocity ratios *l*/*v* = 10,20,40,40,50ms (see legend). The peak amplitude of the curves decreases with increasing halfsize-to-velocity ratio, but also with increasing *a* and *p*, respectively (not shown). (**c**) Identical with figure panel *b*, but here all curves 𝒮(*g*_3_) were re-scaled to the range from 0 to 1. For comparison, the corresponding *η*-functions are drawn alongside with dashed lines (*α* = 4.7, *δ* = 0, and *t*_*c*_ = 0.5s in all figure panels).

**Fig 2:**
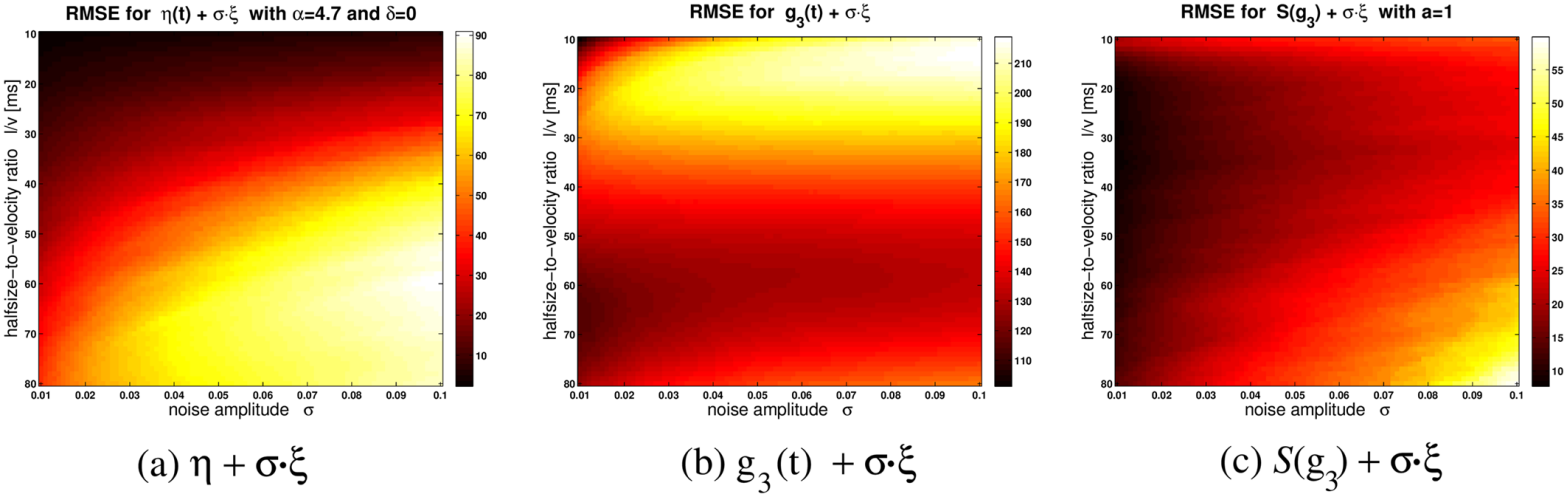
How noise interacts with maximum detection. This figure shows how the numerical detection of the global maximum ${\hat{t}}_{\text{max}}$ is affected by adding noise to the *η*-function, *g*_3_(*t*), and 𝒮(*g*_3_). To this end, the root mean square error (RMSE) between ${\hat{t}}_{\text{max}}$ and the theoretical (i.e. noise-free) *t*_max_ is determined across 999 trials. The RMSE is computed as a function of noise amplitude *σ* (abscissae) and the halfsize-to-velocity ratio *l*/*v* (ordinates). Brighter (“hotter”) colors denote bigger RMSE values (see colorbar; units in milliseconds). Noise is added as follows. Let *ξ* be a normal-distributed random variable with mean zero and standard deviation one. Then, (**a**)*η*(*t*) + *σξ* is the “noisified” *η*-function (with *δ* = 0 and *α* = 4.7), (**b**)shows the RMSE for *g*_3_(*t*) + *σξ*, and (**c**) 𝒮(*g*_3_) + *σξ* (with *a* = 1 in Eq (8)). The curves of *η*(*t*), *g*_*p*_(*t*) and 𝒮(*g*_*p*_) become flatter for higher halfsize-to-velocity ratios (cf. Fig 1). In addition, the curves of *g*_*p*_(*t*) become flatter for increasing *p*, and the curves of 𝒮(*g*_*p*_) get flatter for *decreasing* values of *a* and *p*, respectively. Flatter curves are associated with an increased RMSE. Although *g*_3_(*t*) + *σξ* is associated with higher RMSE values than *η*(*t*) + *σξ*, the completely “decoded” function 𝒮(*g*_3_) has a better overall robustness against additive Gaussian noise than *η*(*t*). For each value of *l*/*v*, *g*_3_(*t*) and 𝒮(*g*_3_) were re-scaled before noise was added, in order to match the range of *η*(*t*), and thus normalize the RMSE. For all figure panels, *t*_*c*_ = 0.5s and *l* = 0.06m.

If the exponent *p* is fixed, then the biophysical implementation of *o* + *g*_1, 3, 5, …_ and *o* − *g*_2, 4, 6, …_, respectively, should be feasible. Nevertheless, a general drawback of “undoing” the logarithmic transformation with a power law would be that the maximum of the LGMD membrane potential is strongly flattened: The bigger *p* is, the more (Fig 1a). As a consequence, the reliability of detecting the time of peak firing drops already for small noise amplitudes (Fig 2b). The different phases of escape jumps seem to occur around the peak firing rate of the DCMD [25], although this does not automatically imply that the peak time is explicitly decoded by the motor system of the locust. Experimentally measured timings of DCMD peak firing have standard deviations of ≈ 50ms [20]. The comparatively high variability of the response maximum as predicted by *g*_3_(*t*) (Fig 2b) stands in contrast to these observations, since standard deviations are higher than 100ms even for small noise levels. It seems therefore that the sole power law of Eq (6) cannot reproduce LGMD firing patterns. Is it possible to regain the undistorted, “LGMD-like” response curves from *g*_*p*_(*t*)? Reference [17] holds a general mathematical solution in order to map the *η*-function to the firing rate of the LGMD. The mapping function is a (here simplified) sigmoid 𝒮(*x*) = [1 + exp(− *ax*)]^−1^. The value of *a* determines whether the activation from zero to one proceeds in a more linear way (*a* ≪ 1) or according to step-function (*a* ≫ 1). With *g*_*p*_ = (log*η*)^*p*^ Eq (6) one obtains

$$\begin{array}{c} 𝒮\left(g_{p}\right)=\frac{\eta^{a{\left(\log\eta \right)}^{p-1}}}{1+\eta^{a{\left(\log\eta \right)}^{p-1}}} \end{array}$$ (8)

and specifically 𝒮(*g*_1_) = *η* ^*a*^/(1+*η* ^*a*^). With an appropriate choice of the parameters *a* and *p*, respectively, the function 𝒮(*g*_*p*_) reduces the initial activity compared to the ordinary *η*-function. In this way the peak is more pronounced (see Fig 1c). However, with increasing *p* and *a*, respectively, the peak amplitudes of curves with higher *l*/*v* decrease relative to low halfsize-to-velocity ratios (see Fig 1b). The peak values for a set of *l*/*v* values could quickly span several orders of magnitude for already “moderate values” *a* > 1 (e.g. *a* = 5) and *p* > = 1.

What are the possible implications for biology of the mathematical exercise presented in this section? First, a direct biophysical implementation of *η* (with logarithmic decoding based on a power law) seems to be insufficient. At least mathematically, an additional (probably nonlinear) function is required (e.g. based on Eq (8)). In reference [17], it has been demonstrated that the linearity constraint Eq (2) implies a partial differential equation of the structure *x*(∂*f*/∂*x*) = −(∂*f*/∂*y*) with a general solution *f* = *h*(*x* exp(− *y*)). This means that *η* is defined up to a (monotonically increasing) function *h* that “characterizes the transformation between *η* and the firing rate of the LGMD” [17]. In [17] the response curves of the LGMD were fitted with a sigmoidal function whose parameters depended on the halfsize-to-velocity ratio and also on the rising phase (i.e. a sigmoid fitted to the curve until the response peak at *t*_max_) and falling phase (a second sigmoid fitted to the curve after *t*_max_). Nevertheless, to the best of my knowledge, this nonlinearity has not been identified biophysically so far. This prompted me to formulate an alternative model, which is presented below.

### “*n-ψ*”—a model of the LGMD neuron

In this section, we introduce a new model (“*n-ψ*”) for the LGMD and other collision-sensitive neurons with *η*-like response. While *n-ψ* is biophysically more plausible than *η*, it is not a biophysically *detailed* model (compared with, e.g. [22]). The *n-ψ*-model is based on its predecessor model Ψ, which used an exact power law for re-scaling the inhibitory input [19]. An important component of *n-ψ* is noise: *n-ψ* generates an approximative power law by pooling of noisy and thresholded inhibitory inputs. The power law is only approximative because it turns to a linear relationship sufficiently far from threshold. The pooling process may be implemented *in vivo* by integrating synaptic inputs across the dendritic tree. *n-ψ* is based on a standard RC-circuit describing the membrane potential *V* of the LGMD neuron [26]

$$\begin{array}{c} C_{m}\frac{dV}{dt}=\beta (V_{rest}-V)+g_{exc}(V_{exc}-V)+g_{inh}(V_{inh}-V)\;. \end{array}$$ (9)

As we do not use a spiking mechanism, we assume that [*V*]^+^ ≡ max(*V*, 0) (the half-wave-rectified membrane potential) directly represents the LGMD’s mean firing rate. (For simplicity, we will omit physical units in what follows). The membrane capacitance *C*_*m*_ is set to unity in all simulations; *β* ≡ 1/*R*_*m*_ is the leakage conductance across the cell membrane and *R*_*m*_ is the membrane resistance; *g*_*exc*_ ≥ 0 and *g*_*inh*_ ≥ 0 are the excitatory and inhibitory synaptic inputs. The corresponding reversal potentials *V*_*exc*_ and *V*_*inh*_, respectively, represent upper (*V*_*exc*_ > *V*_*rest*_) or lower (*V*_*inh*_ < *V*_*rest*_) limits to *V* if the neuron is driven by the associated input *g*_*exc*_ or *g*_*inh*_. The reversal potentials are asymptotically approached for sufficiently high excitatory or inhibitory drive. Typically one sets *V*_*inh*_ ≤ *V*_*rest*_ ≤ *V*_*exc*_, what implies *V*_*inh*_ ≤ *V*(*t*) ≤ *V*_*exc*_. Shunting inhibition is defined by setting a reversal potential equal to the resting potential *V*_*rest*_. Shunting inhibition essentially increases the leakage conductance, and thus becomes effective only when the neuron is driven away from *V*_*rest*_. The time scale of Eq (9) is determined by the membrane time constant *τ*_*m*_ ≡ *C*_*m*_/*β*. If the synaptic inputs vary on a slower time scale than *τ*_*m*_, then the neuron will reach its equilibrium potential *V*_∞_ at each instant *t* according to *V*(*τ*) = *V*_∞_(1 − *exp*(− *τ*/*τ*_*m*_)), where *V*_∞_ is defined as:

$$\begin{array}{c} V_{\infty }\equiv \frac{V_{rest}\beta +V_{exc}g_{exc}+V_{inh}g_{inh}}{\beta +g_{exc}+g_{inh}}\;. \end{array}$$ (10)

Without synaptic input, *V* approaches *V*_*rest*_ with *τ*_*m*_. Note that with *C*_*m*_ = 1, the time scale is set directly by *β*, where the synaptic inputs are subjected to a higher degree of lowpass filtering for lower values of *β*. Slowly varying inputs *g*_*exc*_, *g*_*inh*_ > 0 modify the time scale approximately to *τ*_*m*_/(1 + (*g*_*exc*_ + *g*_*inh*_)/*β*). With highly dynamic inputs, *τ*_*m*_ also varies with time.

For the definition of synaptic input we first introduce the low-pass filtered angular size *ϑ*(*t*) and angular velocity $\dot{\vartheta }(t)$, respectively (see section S8 in [21] for a brief introduction):

$$\begin{array}{c} \vartheta \left(t+\Delta t_{stim}\right)=\zeta_{0}\vartheta \left(t\right)+\left(1-\zeta_{0}\right)\Theta \left(t\right) \end{array}$$ (11)

$$\begin{array}{c} \dot{\vartheta }\left(t+\Delta t_{stim}\right)=\zeta_{1}\dot{\vartheta }\left(t\right)+\left(1-\zeta_{1}\right)\dot{\Theta }\left(t\right)\;. \end{array}$$ (12)

Low-pass-filtering is supposed to model delays and filtering effects as they are introduced by the pre-synaptic layers (lamina and medulla). The stimulation time scale is fixed by Δ*t*_*stim*_, and 0 ≤ *ζ*_*i*_ < 1 (*i* = 1,2) determines the degree of low-pass filtering (no filtering would take place for *ζ*_*i*_ = 0). The lowpass filtered signals lag behind the original signals (the bigger *ζ*_*i*_, the more). This behavior is important to explain experimental observations, where the LGMD’s activity continues to rise after ttc [14, 18, 19]. Lowpass filtering also reduces stimulus-dependent noise in the optical variables (Θ and $\dot{\Theta }$, Eqs (3) and (4), repsectively), particularly when the angular size is small [21]. The *n-ψ*-model is defined by assigning the following synaptic inputs:

$$\begin{array}{c} g_{exc}\left(t\right)=\dot{\vartheta }\left(t\right) \end{array}$$ (13)

$$\begin{array}{c} g_{inh}\left(t\right)=\frac{\gamma }{N}\sum_{i=1}^{N}{\left[\vartheta \left(t\right)+\sigma \cdot \xi_{i}-\Delta_{0}\right]}^{+}\;. \end{array}$$ (14)

**Fig 3:**
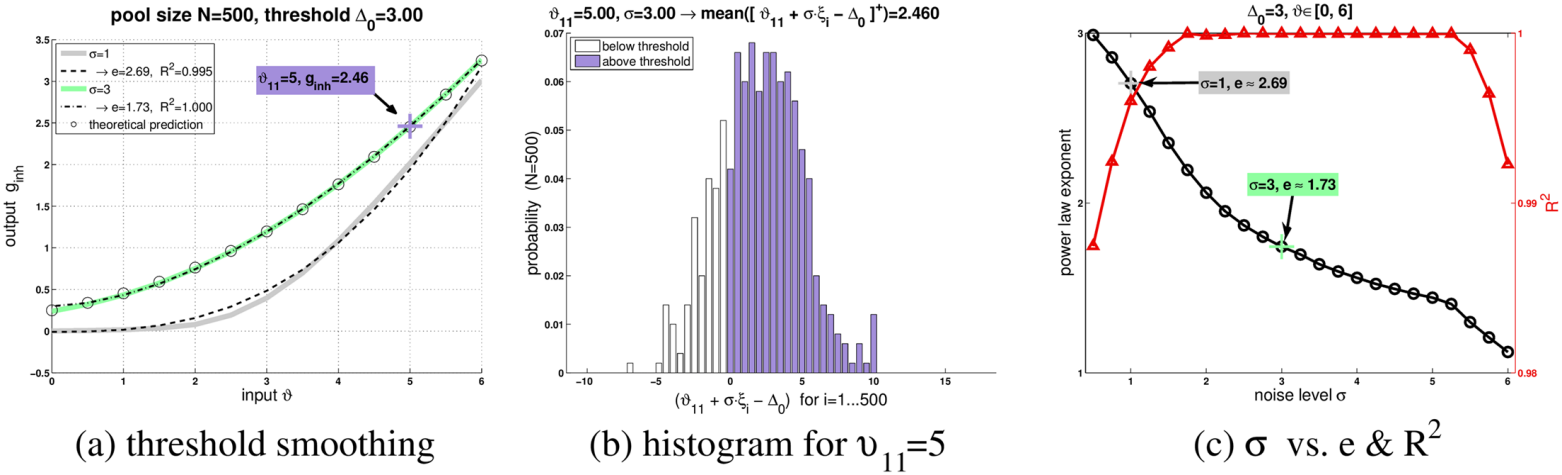
Threshold smoothing and power law (illustration of Eq (14)). (**a**)The dependence of *g*_*inh*_ on 13 values of *ϑ* is shown at two different noise levels (*σ* = 1 and *σ* = 3, see legend) along with respective fit of a power law (exponents *e* = 2.69 and *e* = 1.73, see legend). For *σ* = 3, the theoretical prediction is shown as well (circles; cf. Equation A7 in S1 Text) (**b**)For *ϑ*_11_ = 5 (eleventh data point), the distribution of *ϑ*_11_ + *σ* ⋅ *ξ*_*i*_ − Δ_0_ is shown from *i* = 1 to *i* = *N*. In order to compute *g*_*inh*_, these values are first half-wave rectified (what zeros the values which correspond to the white bars in the histogram), and subsequently averaged (non-zero contribution from values represented by the colored bars). This eventually yields *g*_*inh*_ = 2.46 for *ϑ*_11_. Thus, non-zero *g*_*inh*_-responses for some *ϑ* < Δ_0_ result from fluctuations with *ξ*_*i*_ > 0 such that *ϑ* + *ξ*_*i*_ > Δ_0_. If *ϑ* is decreased, then whole distribution will shift to the left. (**c**) Power laws were fitted to the *g*_*inh*_ versus *ϑ* curves and exponents *e* (circles) along with *R* ^2^ (triangles) are plotted. These curves are well described by a power law only at intermediate noise levels, here 1.75 ≤ *σ* ≤ 5.25.

Where [⋅]^+^ = max(⋅,0). Similar to *η* Eq (1), *n-ψ* uses lowpass-filtered angular size for excitation. Unlike *η*, however, *n-ψ* does not use an exponential function but instead sums threshold processes. The threshold is set by Δ_0_. The *ξ*_*i*_ are random numbers drawn from the standard normal distribution (Fig 3b). Thus, *σ*⋅*ξ*_*i*_ adds Gaussian noise with standard deviation *σ* to *ϑ*. We furthermore require that *corr*(*ξ*_*i*_, *ξ*_*j*_) = *δ*_*ij*_, where *corr*(⋅) denotes correlation, and *δ*_*ij*_ is the Kronecker delta. The number of threshold processes that are pooled is *N*, and *γ* is a synaptic weight.

### Biophysical motivation for *g*_*inh*_

The LGMD’s dendritic tree has one excitatory field and two smaller inhibitory ones. It receives about 15,000 retinotopically organized excitatory inputs from the medulla [22]. Each of the inhibitory fields is contacted by some 500 axons originating in the second optic chiasm [27]. Response dynamic and response characteristics are similar in both inhibitory pathways. They can be distinguished, however, by contrast polarity. The ON-pathway responds to increments in luminance, and the OFF-pathway responds to luminance decrements. Along the excitatory visual pathway (ommatidia, lamina, medulla and lobula), the signal-to-noise ratio decreases, being lowest in the LGMD [4]. In Eq (14), we assume that noise is present in the inhibitory pathway as well, and that it is uncorrelated for each of the *N* = 500 inhibitory inputs (“processes”) that are pooled by the LGMD. For the sake of clarity we did not incorporate noise in *g*_*exc*_ Eq (13), because the predictions made by *n-ψ* would not change much if we did so (Section B in S1 Text). Conversely, *n-ψ* would fail to predict the LGMD’s response characteristics with noise in the excitatory pathway but without noise in *g*_*inh*_. Since the pooling of noisy threshold processes approximates a power law, this behaviour of *n-ψ* is consistent with the schematic proposed in [22], where both the excitatory and the inhibitory input are scaled according to a power law (with exponents 2–3 and 2, respectively).

The *integrated* (or pooled) inhibitory input to the LGMD is supposed to encode angular size [27, 28]. But Eq (14) rather suggests that *ϑ* is identical for *each* inhibitory process, and that only the noise varies from one input to the next. To see that this is nevertheless a reasonable simplification, assume that the (noise-free) *ϑ*(*t*) at each instant *t* is just the sum of many individual *ϑ*_*i*_(*t*), that is *ϑ*(*t*) = ∑_*i*_ *ϑ*_*i*_(*t*), with 1 ≤ *i* ≤ *N*. The index *i* could be retinotopically arranged, but this is not relevant. Then, Eq (14) would read [*ϑ*_*i*_(*t*) + *σ* ⋅ *ξ*_*i*_ − Δ_0_]^+^, what looks more decent. The problem is that we do not know the exact distribution of the *ϑ*_*i*_(*t*). Therefore we suppose that it is uniform, meaning that all *ϑ*_*i*_(*t*) contribute equally. But then, *ϑ*(*t*) ≡ *Nϑ*_*i*_(*t*) if *ϑ*_*i*_(*t*) = *ϑ*_*j*_(*t*) for all *i*, *j*. Thus, with uniformly distributed *ϑ*_*i*_(*t*), we can draw 1/*N* outside the rectification, rescale the noise level *σ* and the threshold Δ_0_ by *N*, and readily arrive at Eq (14).

### Threshold smoothing

The threshold operation [*x*]^+^ = max(*x*, 0) is zero for *x* ≤ 0 and *x* otherwise. Around *x* = 0, we have a discontinuity. If *x* is set to a suitable negative number, then by adding noise *σ* ⋅ *ξ* to *x* we may obtain non-zero responses. The probability of noise-elicited threshold crossing will increase as *x* approaches zero. By computing a mean value ∝ ∑_*i*_[*x* + *σ* ⋅ *ξ*_*i*_]^+^ across many channels at some instant in time, one may effectively smooth out the discontinuity at the threshold and create continuous responses (“threshold smoothing”: In ref. [10] averaging was carried out across successive trials). Fig 3a shows two representative curves *g*_*inh*_ vs. *ϑ* for the noise levels *σ* = 1 and *σ* = 3. The threshold is fixed at Δ_0_ = 3. Bigger *σ* mean more noise, what in turn causes a higher degree of threshold smoothing, since non-zero *g*_*inh*_-responses will be obtained already for *ϑ* < Δ_0_. For *ϑ* > Δ_0_, *g*_*inh*_ gradually approaches a linear response. Around Δ_0_, a power law provides a good description of *g*_*inh*_ [11, 12]. The two curves in Fig 3a were fit by power laws with exponents *e* = 2.7 and *e* = 1.7, respectively. Fig 3c (circles) illustrates that higher noise levels result in smaller exponents. Nevertheless, a power law is only adequate within a certain range of *σ*: the red curve in Fig 3c (triangles) representatively shows the *R* ^2^ as a goodness of fit measure. The highest *R* ^2^ values are obtained around 1.75 ≤ *σ* ≤ 5.25. For smaller *σ*, the response curves show less smoothness, while for bigger *σ*, *g*_*inh*_ depends “more linearly” on *ϑ*. Mathematically, it can be shown that *g*_*inh*_ is composed of three additive terms (Equation A7 in S1 Text): A linear term plus an error function plus a Gaussian [11, 12]. As a function of *ϑ*, the error function and the Gaussian will only vary within a certain interval around Δ_0_. Outside this interval, the Gaussian approaches zero and the error function approaches a constant value.

### Predicting the response characteristics of the LGMD neuron

LGMD responses have two important properties: A response maximum before collision on the one hand [16], and linearity on the other [17]. The *η*-function and *n-ψ*’s predecessor model Ψ successfully predicts these properties (Methods Section). Below we study the corresponding predictions of *n-ψ*.

**Fig 4:**
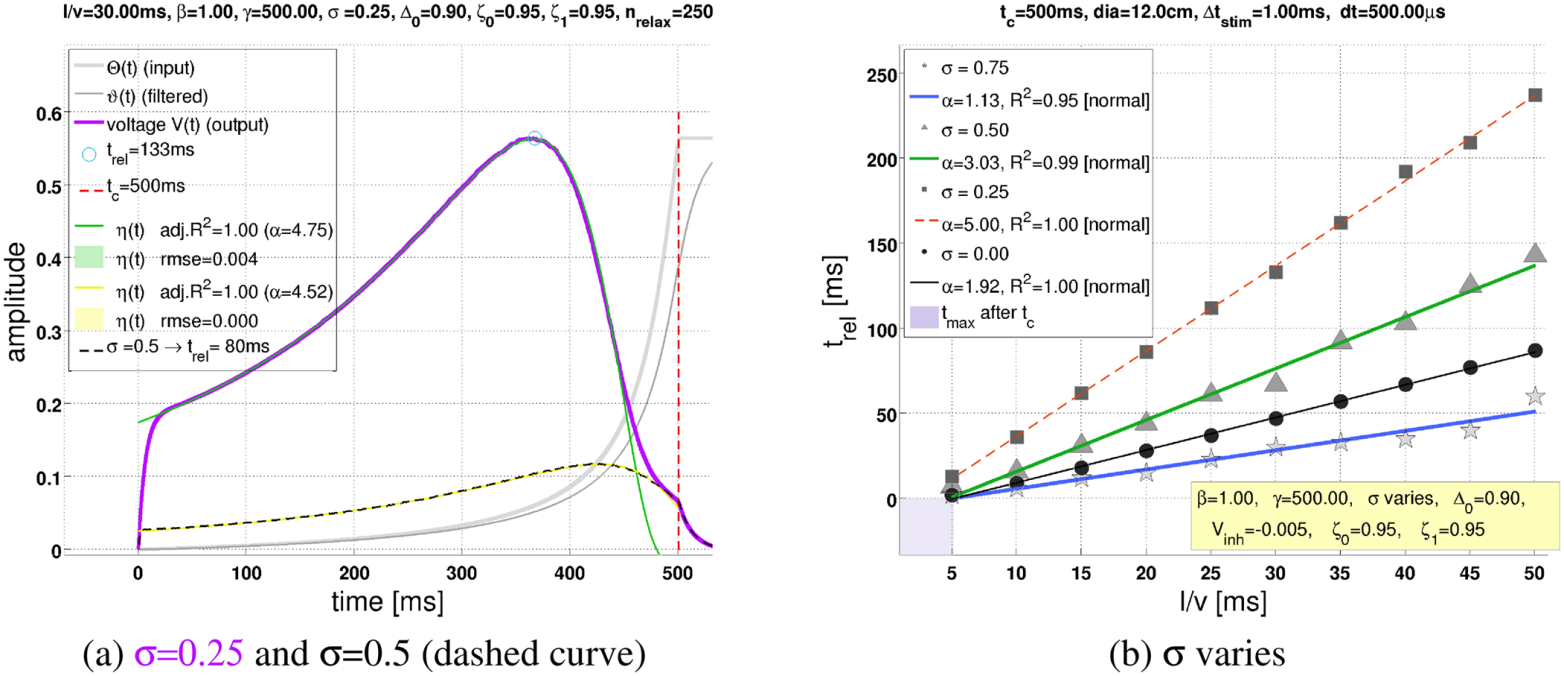
Influence of the inhibitory noise level *σ*. (**a**)The figure illustrates how an increase of the inhibitory noise level from *σ* = 0.25 to *σ* = 0.50 moves the maximum of *n-ψ* towards *t*_*c*_ = 500ms, thus *t*_rel_ (= the remaining time to collision after the peak) decreases from 133ms to 80ms. Furthermore, the amplitude of the *n-ψ*-response decreases. (**b**)The line fits represent the linear dependence of *t*_rel_ from *l*/*v*, where different symbols and line colors, respectively, correspond to different values of *σ*. The smallest line fit slopes *α* see Eq (2) were obtained for *σ* = 0 and *σ* = 0.75, that is in the absence of inhibitory noise (*α* = 1.92) and for the highest tested noise level (*α* = 1.13). For the intermediate noise levels *σ* = 0.25 and *σ* = 0.50, respectively, the biggest slopes were measured (cf. figure legend; “normal” means that the residuals were normal distributed according to a one sample Kolmogorov-Smirnov test).

**Fig 5:**
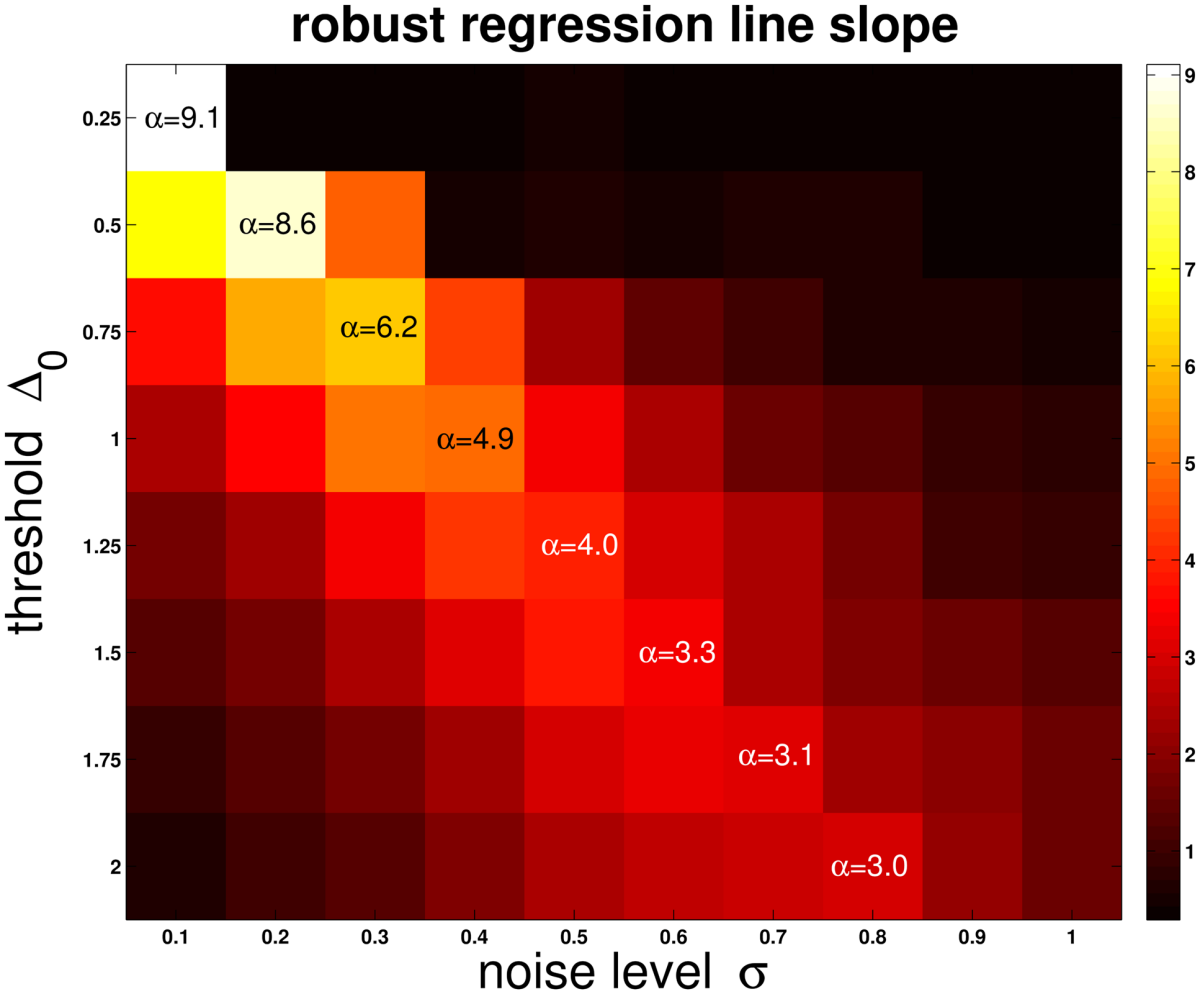
Line fit slopes. This figure shows how the line fit slopes *α* depend on *σ* and Δ_0_. The proceeding is similar to Fig 4b (where Δ_0_ was fixed): For each *σ* and Δ_0_, *t*_rel_ was computed as a function of *l*/*v*. A line was then fit to these data (with a robust fit algorithm), and its slope *α* was recorded. If Δ_0_ is held constant and we consider *α* as a function of *σ* only, then this function adopts a maximum slope. The maxima are situated along a crest, with the corresponding values being indicated in the figure. Hotter colors indicate higher slopes.

Fig 4a shows that *n-ψ* has an activity maximum. When the noise level *σ* is increased (while keeping the threshold Δ_0_ constant), then the maximum shifts towards *t*_*c*_, and the amplitude decreases. Fig 4b confirms the linear dependence of *t*_rel_ on *l*/*v* for the *n-ψ*-model Eq (2). The noise level *σ* is decisive for the slope *α* of the corresponding line fits. Interestingly, *α* adopts a maximum value at some noise level. Fig 5 shows the dependence of *α* on *σ* and Δ_0_: The location of the maximum *α* as a function of *σ* depends also on Δ_0_. Bigger Δ_0_ mean that the maximum *α* is obtained at smaller *σ*. Furthermore, the maximum value of *α* will be higher if Δ_0_ is bigger.

One can ask how the predictions of *n-ψ* are influenced by excitatory noise. As it turns out, excitatory noise distorts the response curves of *n-ψ* somewhat, and shifts the response maxima slightly towards *t*_*c*_ (Fig. A1 in S1 Text). Nevertheless, excitatory noise has only a negligible effect on *α*, and thus does not alter the linear relationship between *t*_rel_ and *l*/*v*. (More details are provided in Section B in S1 Text).

**Fig 6:**
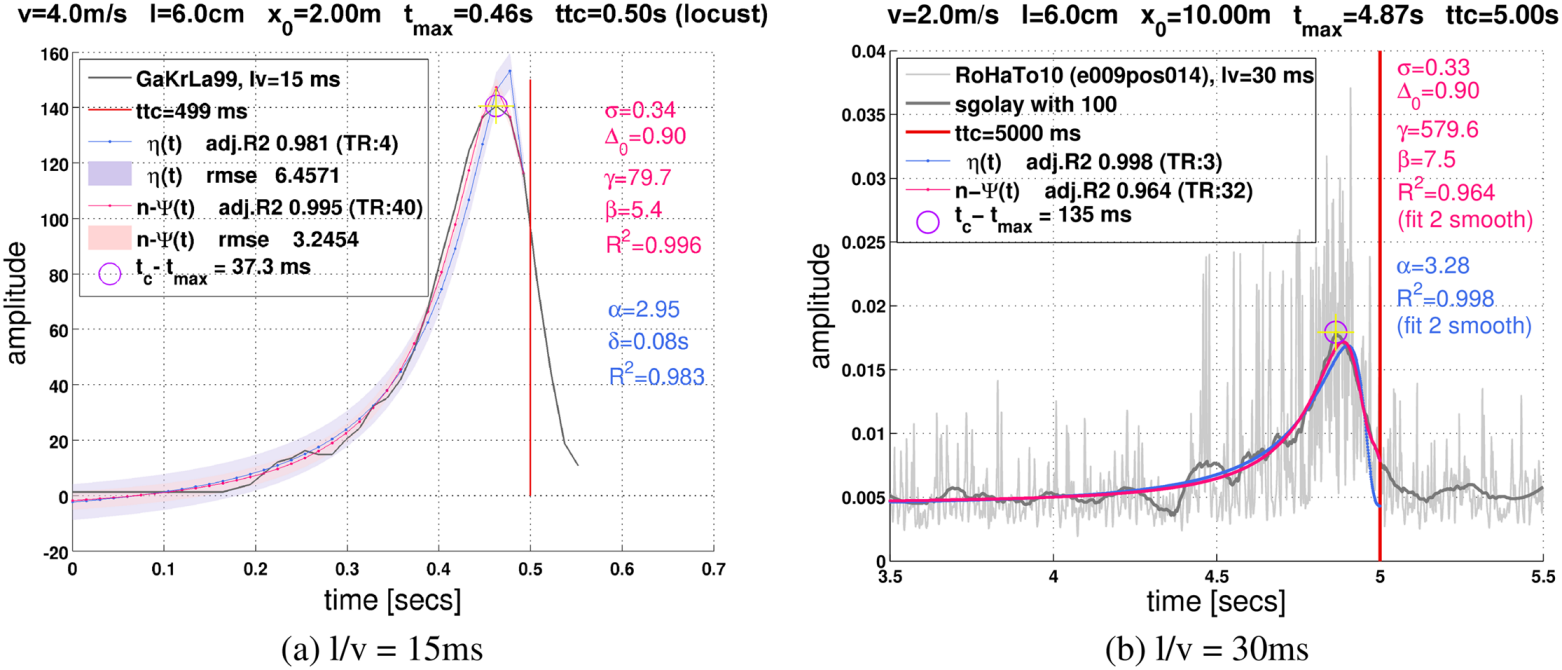
Prediction of LGMD responses by *n-ψ*. (**a**)The figure shows the data from figure 3 of reference [17] (locust), where looming dark squares were used for stimulation (*t*_*c*_ = 0.5s: vertical red line). The *η*-function and *n-ψ* perfectly match these data. More examples are shown in Section D.5 in S1 Text. (**b**)DCMD spike trace in response to an approaching black square (*t*_*c*_ = 5s, *l*/∣*v*∣ = 30ms, ref. [29]) directed to the eye center of a gregarious locust (final visual angle 50^*o*^). Data show the first stimulation so habituation is minimal. The spike trace (sampled at 10^4^Hz) was full wave rectified, lowpass filtered, and sub-sampled to 1ms resolution. Firing rate was estimated with Savitzky-Golay filtering (“sgolay”), and fit by the *η*-function and *n-ψ*, respectively. Both functions fit the firing rate estimates very nicely. More details and further examples are provided in Section D.6 in S1 Text.

How does *n-ψ* compare to the *η*-function when it comes to fitting response curves of the LGMD? We resampled 36 corresponding curves from the figures of several publications to this end [21], and fitted the two functions to them. Fig 6a illustrates that *η* and *n-ψ* describe the LGMD responses very well (all fits are shown in Section D.5 in S1 Text). Excellent predictions are also obtained for non-trial-averaged recording traces (Fig 6b; more examples are shown in Section D.6 in S1 Text). The 36 response curves were fit, on the average, with a median noise level *σ* = 0.4 ± 0.1 (Fig. D3a in S1 Text), and a median threshold Δ_0_ = 0.9 ± 0.1 (Fig. D3b in S1 Text). These values should be compared with the median *α* = 3.1 ± 0.7 (D2a in S1 Text) of the *η*-function, and the exponent *e* = 2.7 ± 0.5 of the *ψ*-model (Fig. D2b in S1 Text).

**Fig 7:**
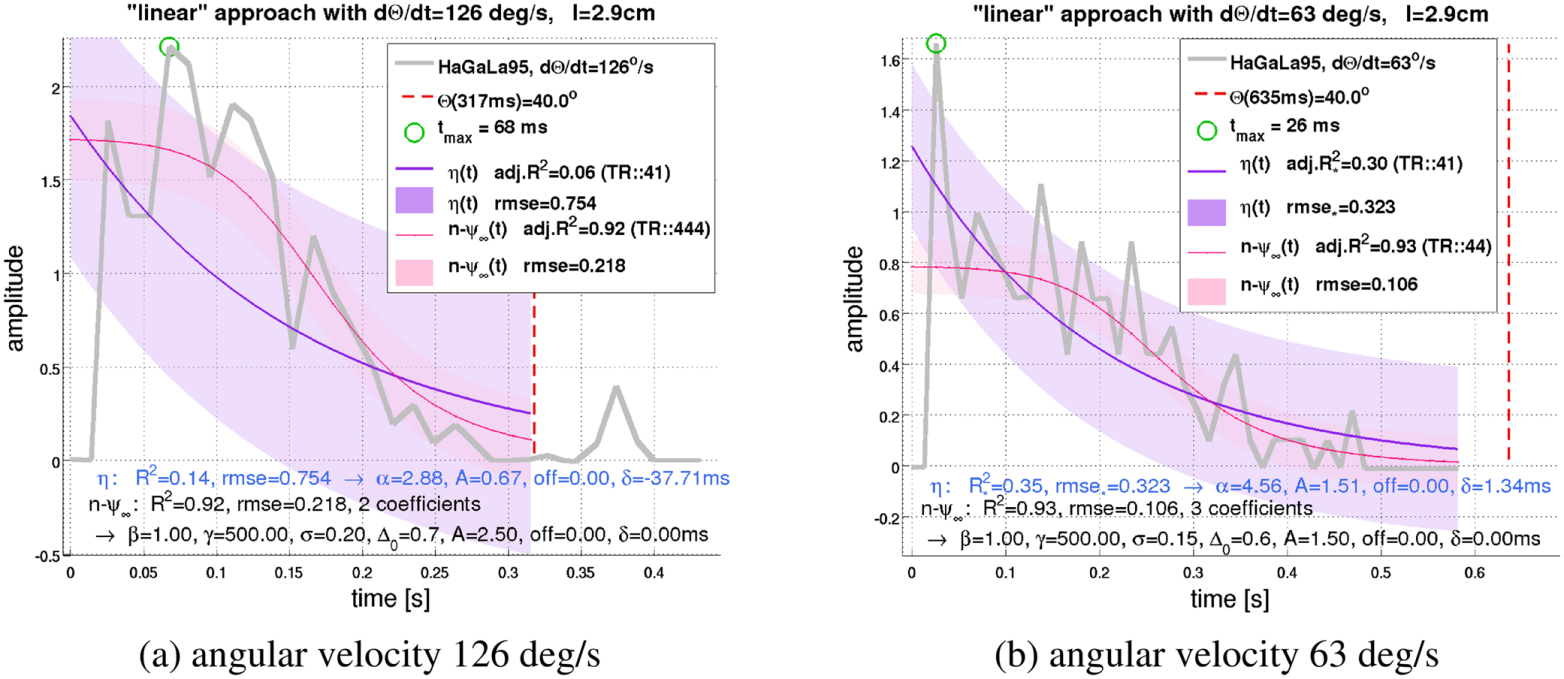
LGMD stimulation with constant angular velocity. The original data (legend label “HaGaLa95”) were resampled from ref. [16] and show DCMD responses to an object approach with $\dot{\Theta }=const$, what is equivalent to that Θ increases linearly with time. The *η*-function (fitting function: *A* *η*(*t* + *δ*) + *o*) and *n-ψ* Eq (10) were fitted to these data (see Section D in S1 Text for specifics). (**a**)(Figure 3 Di in [16]) Good fits for *n-ψ* are obtained with *σ* = 0.2 and Δ_0_ = 0.7. *n-ψ* follows a sigmoid-like curve that (subjectively) appears to fit the original data better than *η*. The fitting parameters for *η* were {*A*, *α*, *δ*, *o*} (“*TR:41*”), and {*σ*, Δ_0_} for *n-ψ*(“*TR:444*”). (**b**)(Figure 3 Dii in [16]) The fit of *n-ψ* agrees excellently with the data for *σ* = 0.15 and Δ_0_ = 0.6. The fitting parameters for *n-ψ* were {*β*, *σ*, Δ_0_} (“*TR:44*”). Figure D26 in S1 Text shows how the linear approach data could also be predicted by explicitly integrating *n-ψ* instead of fitting the equilibrium solution.

A “recession” is a stimulation protocol where the object moves away from the eye—just the opposite to an object approach. DCMD responses to receding objects are significantly smaller than those to approaching objects [14]. Once an object starts to recede, the DCMD response reaches an activity maximum, which is smaller than that of a corresponding approach. Subsequently, the activity decreases quickly. Thus, the directional sensitivity in DCMD responses is reflected in overall activity, but also in terms of curve shape. The *η*-function predicts symmetric responses (approach vs. recession) [16]. The *n-ψ*-model, on the other hand, predicts an asymmetry of 40*ms* in the timing of the response peaks for the configuration of Fig 4a, because of lowpass filtering. A perhaps more interesting kind of stimulation is shown in Fig 7, where $\dot{\Theta }=const$. A constant angular velocity implies that angular size is linearly increasing with time. The experimentally obtained response curves of the LGMD to this “linear approach” are nevertheless decreasing [16]. In that case, *n-ψ* provides a much better description of the LGMD data than the *η*-function, both in terms of goodness-of-fit measures and subjective judgement (“Chi-by-eye”).

Experimental evidence [23] and modelling [22] suggest a logarithmic scaling of angular velocity $\dot{\Theta }$, which provides the excitatory input to the LGMD. The *n-ψ*-model also predicts such logarithmic encoding of $\dot{\Theta }$ mainly as a consequence of the reversal potential *V*_*exc*_ > *V*_*rest*_ in Eq (9). When the excitatory input *g*_*exc*_ increases, then the driving potential *V*_*exc*_ − *V*(*t*) decreases. Thus, *V*_*exc*_ represents an upper limit to *V*(*t*), which is approached asymptotically. The corresponding (saturating)“approach curve” *V*(*g*_*exc*_) is approximately logarithmically. This mechanism is identical to the one proposed in [22].

## Discussion

This paper introduces the *n-ψ*-model for a class of collision-sensitive neurons that have an activity maximum before collision in response to object approaches with constant velocity. Noise plays a key role for *n-ψ*, because it approximates a power law in the inhibitory pathway by means of threshold smoothing Eq (14). Threshold smoothing is predicted as a consequence of integrating many inhibitory inputs (“channels”). The integration process (or pooling) could be carried out, for example, by the dendritic tree of the *Lobula Giant Movement Detector* neuron (LGMD). The only preconditions which have to be met for each channel are the presence of noise (uncorrelated across channels), and a response threshold. As a consequence, *n-ψ* accounts successfully for many response properties of the LGMD, while at the same time it offers a good balance between biophysical plausibility, computational complexity, and the physical parameters of collision: *n-ψ* takes angular size and angular velocity as input (physics), and connects them with each other by an equation for the LGMD’s membrane potential (biophysics, Eq (9)).

**Fig 8:**
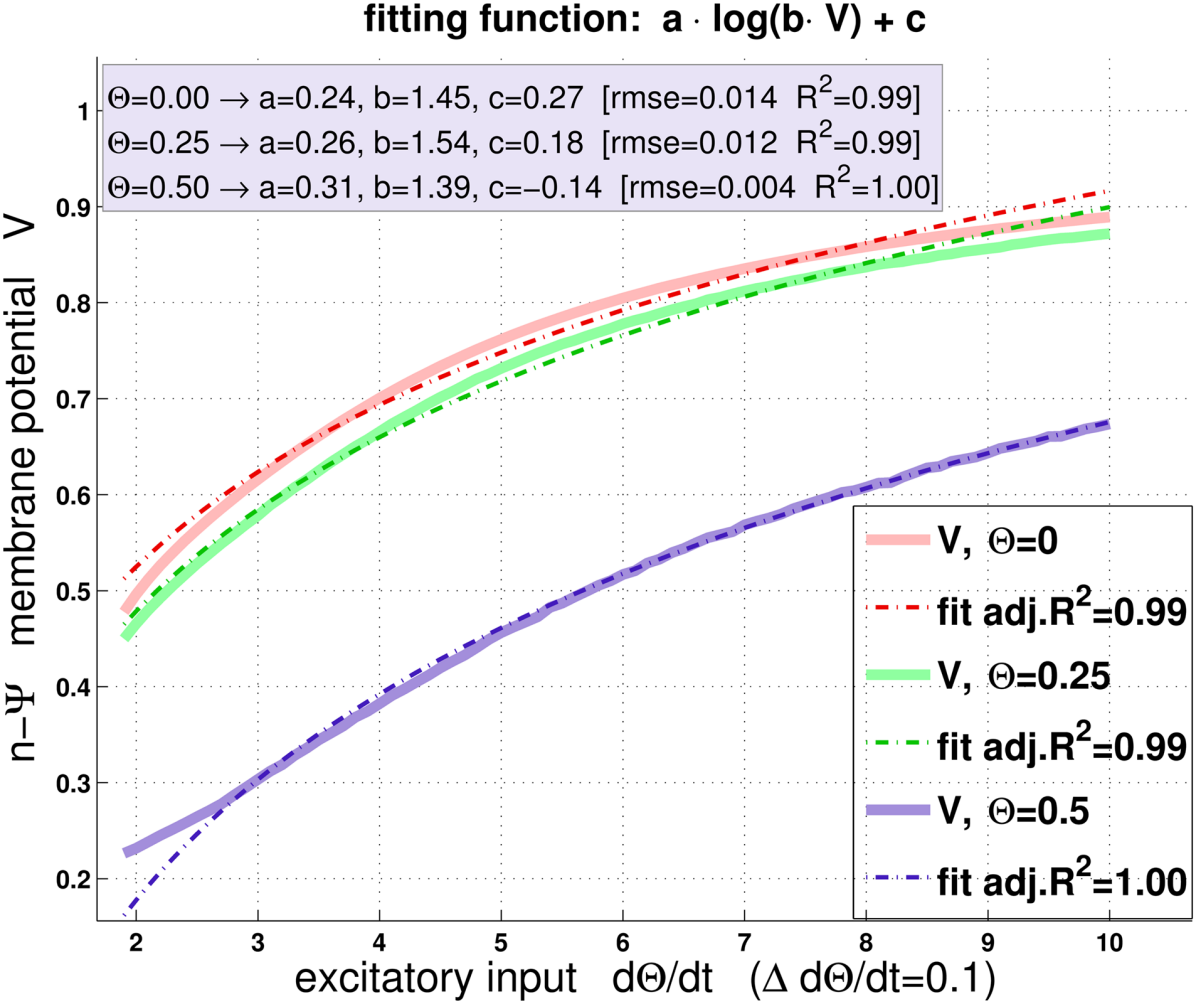
The *n-ψ*-model is consistent with logarithmic compression. A logarithmic compression of rate of expansion has been shown in refs. [22, 23]. Although logarithmic compression is not explicitly “used” as a computational feature in *n-ψ*, it nevertheless appears as a “by-product”: The light-colored solid curves show Eq (9) as a function of angular velocity $\dot{\Theta }$ (= excitatory input) for three constant values of angular size Θ (= inhibitory input). Notice that because of *V*_*inh*_ ≈ *V*_*rest*_ in Eq (9), increasing inhibition alone would be without effect (see Methods Section). The membrane voltage *V* of *n-ψ* could be reasonably well fit by the logarithmic function *a*log(*bV*) + *c* in the considered range of $\dot{\Theta }$ (dashed lines). The values from $\dot{\Theta }=0$ to $\dot{\Theta }=2$ were excluded from fitting, due to initial transients. For computing *V*, $\dot{\Theta }$ was incremented linearly in steps of 0.1. Goodness-of-fit measures and fitting parameters are shown in the figure.

How do other models for the LGMD compare to *n-ψ*? The *η*-function requires multiplication and an exponential function, $\eta =\dot{\Theta }\times \text{exp}(-\alpha \Theta )$ [16], and predicts many of the response characteristics of the LGMD. For *η*, the only mechanism for explaining that the response peak of the LGMD could occur *after* projected collision is the temporal delay *δ* in conjunction with small halfsize-to-velocity ratios. The predecessor model of *n-ψ* predicts such situations as a consequence of “extreme” stimulation conditions [19]. Compared to *n-ψ*, the *η*-function represents furthermore a poor fit to the linear approach data shown in Fig 7. Moreover, the formal arguments that were exposed above suggest that the proposed biophysical implementation of *η* (according to [23]) seems to be incomplete. Note that the *n-ψ*-model predicts the logarithmic encoding of angular velocity as a by-product (Fig 8). In contrast to *η*, however, this does not imply a direct (as opposed to an emergent) implementation of multiplication in the LGMD [19].

The LGMD has also been modelled with neuronal networks, which consist typically of several interacting presynaptic layers (e.g. [22, 30, 31]; real-world video sequences & robotics: e.g. [32–35]). However, in such network models it is often not straightforward to identify excitatory and inhibitory input to the (model) LGMD with the physical variables of the object approach (i.e. angular size and rate of expansion).

Apart from the response peak, the LGMD has another characteristic feature: The time that remains from the response peak until projected collision *t*_rel_ depends linearly from the object’s halfsize-to-velocity ratio *l*/*v* Eq (2). This characteristic linear relationship is successfully reproduced by *n-ψ* (Fig 4). The slope of corresponding line fits reveals an “inverted-U” behaviour as a function of the noise level *σ*: Maximum slopes are attained for intermediate values of *σ* (Fig 5). Excitatory noise, on the other hand, failed to exert a significant influence on *n-ψ* (Section B in S1 Text).

From the linear relationship of *T*_rel_ = *t*_rel_ − *δ* vs. *l*/*v* (with slope *α* and intercept *δ*), the *η*-function predicts that the activity maximum (of the LGMD) always occurs shortly after (delayed by *δ*) the angular size has reached Θ(*t*_max_) Eq (5). Because Θ_*max*_ = Θ(*t*_max_ + *δ*) depends *only* on *α*, and because the slope depends on *σ* in the *n-ψ*-model (Fig 5), it is possible that noise in the inhibitory pathway is decisive for setting Θ_*max*_. Of course, the rest of the biophysical parameters (like *β*, Δ_0_, and such) all have some influence [19]. If one wished to verify this hypothesis, then it would be necessary to selectively increase or decrease the noise level in the inhibitory pathway, and measure *α*. Noise, however, needs to be injected at the proper site(s), which would have to lie before thresholding and pooling, respectively, takes place.

## Supporting Information

S1 Text **Pooling of Noisy Threshold Units—Mathematical Considerations**
In this section, a closed expression for Eq (14) is derived, which is used for fitting the *n-ψ*-model to neuronal recordings in Section D in S1 Text.
**Noise in the Excitatory Pathway**
In this section the impact of excitatory noise on the predictions of *n-ψ* is studied, where the same threshold smoothing mechanism is used for the excitatory and the inhibitory synaptic input. This is to say that threshold smoothing is applied simultaneously to the excitatory and inhibitory pathway. It turns out that the predictions of *n-ψ* are reasonably robust with this configuration.
**Integration time constant ***dt*** and ***n***_***relax***_**
In this section, the influence of the number of relaxation time steps *n*_*relax*_ and that of the integration time constant *dt* is studied. Specifically, corresponding values of *n*_*relax*_ and *dt* are determined such that *n-ψ* operates close to the equilibrium solution Eq (10). The exact values are important as they were shown to influence the location of the LGMD’s predicted response peak [19].
**Fitting the *n-ψ* and the *η*-Function to Neuronal Recordings**
The *n-ψ*-model is fit to several recording curves from different studies. The fits are juxtaposed with those of the *η*-function. Goodness of fit measures are provided as well, and some fitting results of the predecessor model Ψ are also shown. This section presents the results of previously published studies in the same fitting framework. A common fitting framework enables a meaningful comparison of the respective predictions of the *η*-function and the *n-ψ*-model.
**List of Symbols**
A list with mathematical symbols along with corresponding brief descriptions are provided in this section.
(PDF)
