# Supplementary material for: Dendritic Pooling of Noisy Threshold Processes Can Explain Many Properties of a Collision-Sensitive Visual Neuron
Source: PLoS Comput Biol. 2015 Oct 29;11(10):e1004479. doi: 10.1371/journal.pcbi.1004479 (PMC4626079; doi:10.1371/journal.pcbi.1004479)
Supplement: S1 Text — Pooling of Noisy Threshold Units—Mathematical Considerations In this section, a closed expression for Eq (14) is derived, which is used for fitting the n-ψ-model to neuronal recordings in Section D in S1 Text. Noise in the Excitatory Pathway In this section the impact of excitatory noise on the predictions of n-ψ is studied, where the same threshold smoothing mechanism is used for the excitatory and the inhibitory synaptic input. This is to say that threshold smoothing is applied simultaneously to the excitatory and inhibitory pathway. It turns out that the predictions of n-ψ are reasonably robust with this configuration. Integration time constant dt and nrelax In this section, the influence of the number of relaxation time steps n relax and that of the integration time constant dt is studied. Specifically, corresponding values of n relax and dt are determined such that n-ψ operates close to the equilibrium solution Eq (10). The exact values are important as they were shown to influence the location of the LGMD’s predicted response peak [19]. Fitting the n-ψ and the η-Function to Neuronal Recordings The n-ψ-model is fit to several recording curves from different studies. The fits are juxtaposed with those of the η-function. Goodness of fit measures are provided as well, and some fitting results of the predecessor model Ψ are also shown. This section presents the results of previously published studies in the same fitting framework. A common fitting framework enables a meaningful comparison of the respective predictions of the η-function and the n-ψ-model. List of Symbols A list with mathematical symbols along with corresponding brief descriptions are provided in this section. (PDF) [file pcbi.1004479.s001.pdf]

Supplementary Material to "*Dendritic Pooling of Noisy Threshold Processes Can Explain Many Properties of a Collision-Sensitive Visual Neuron*"

Matthias S. Keil

September 2, 2015

A Pooling of Noisy Threshold Units - Mathematical Considerations

Integral Expression

We pool N channels (or processes) with independent Gaussian noise in each channel,

$$V = \frac{1}{N}\sum_{i=1}^{N}\left[V_0 + \sigma\xi_i - \Delta_0\right]^+ \tag{A1}$$

where $[\cdot]^+ \equiv \max(\cdot, 0)$ denotes half-wave rectification, and Δ_0 is a threshold. The ξ_i are random numbers drawn from the standard normal distribution. It is scaled by σ and shifted by voltage V_0, such that the resulting distribution has an effective mean at $x \equiv V_0 - \Delta_0$ with standard deviation σ.
The result for V from pooling can be calculated by

$$V(x) = \int_{-\infty}^{\infty}\left[\xi p(\xi) d\xi\right]^+ = \frac{1}{\sigma\sqrt{2\pi}}\int_{-\infty}^{\infty}\left[\xi e^{-(\xi-x)^2/2\sigma^2} d\xi\right]^+ \tag{A2}$$

Because of $\exp(\cdot) \geq 0$ we can drop half-wave rectification,

$$V(x) = \frac{1}{\sigma\sqrt{2\pi}}\int_{0}^{\infty}\xi e^{-(\xi-x)^2/2\sigma^2} d\xi = \frac{1}{\sigma\sqrt{2\pi}}\int_{-x}^{\infty}(z+x)e^{-az^2}dz \tag{A3}$$

where we substituted the integration variable $z \equiv \xi - x$ and furthermore defined $a := 1/2\sigma^2$. We split this integral in two parts,

$$V(x) = \frac{1}{\sigma\sqrt{2\pi}}\left(\underbrace{\int_{-x}^{0}(z+x)e^{-az^2}dz}_{\mathcal{I}(x)} + \underbrace{\int_{0}^{\infty}(z+x)e^{-az^2}dz}_{=\sigma^2+x\sigma\sqrt{2/\pi}}\right) \tag{A4}$$

Now, continuing with solving $\mathcal{I}(x)$,

$$\mathcal{I}(x) = \underbrace{\int_{-x}^{0} z e^{-az^2}dz}_{\mathcal{I}_1(x)} + \underbrace{x\int_{-x}^{0} e^{-az^2}dz}_{=\sigma x\,\mathrm{erf}(x/\sigma\sqrt{2})\sqrt{2/\pi}} \tag{A5}$$

where $\mathrm{erf}(x) = \frac{2}{\sqrt{\pi}}\int_0^x \exp(-t^2)dt$ is the error function, and

$$\mathcal{I}_1(x) = \sum_{n=0}^{\infty}\frac{(-a)^n}{n!}\int_{-x}^{0} z^{2n+1}dz = -\sum_{n=0}^{\infty}\frac{(-a)^n}{(2n+2)\cdot n!}x^{2n+2} \tag{A6}$$

where the power series definition of the exponential function was used. For numerical computation, it is sufficient to sum the first terms of the series. Finally, the complete result:

$$
\begin{aligned}
V(x) &= \frac{x}{2}\left(1+\mathrm{erf}(\frac{x}{\sigma\sqrt{2}})\right)+\frac{1}{\sigma\sqrt{2\pi}}\left[\sigma^2-\sum_{n=0}^{\infty}\frac{(-a)^n}{n!}\cdot\frac{x^{2n+2}}{(2n+2)}\right] \quad \text{(A7)}\\
&= \frac{x}{2}\left(1+\mathrm{erf}(\frac{x}{\sigma\sqrt{2}})\right)+\frac{\sigma}{\sqrt{2\pi}}e^{-x^2/2\sigma^2}
\end{aligned}
$$

Where the last equation is from references [2, 3], and can be obtained via a software for symbolic mathematics (e.g. *Mathematica*). The equality of the last two equations was verified by means of computer simulations.

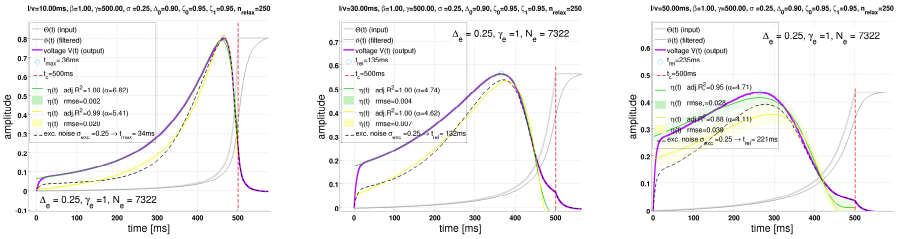

(a) $l/v = 10\text{ms}$ **(b)** $l/v = 30\text{ms}$ **(c)** $l/v = 50\text{ms}$

Supplementary Figure A1 | Illustration of the impact of excitatory noise. The figures show the membrane voltage Equation (6) with noise in the excitatory pathway according to Supplementary Equation (B1) (dashed curve; noise amplitude $\sigma_e = 0.25$: excitatory and inhibitory noise) and without (solid violet curve: only inhibitory noise), for three representative half-size to velocity ratios (**a**) $l/v = 10\text{ms}$; (**b**) $l/v = 30\text{ms}$ (**c**) $l/v = 50\text{ms}$. The time of collision t_c is indicated by a dashed vertical line in each plot. Excitatory noise distorts the n-ψ response curves and shifts their maxima slightly towards t_c (by 2, 3 and 14ms, respectively). With twice the excitatory noise $\sigma_e = 0.50$, the maxima would shift towards t_c by 0, 7 and 19ms, respectively, relative to having no excitatory noise, but only inhibitory noise. Corresponding fits of the η-function are shown as well, with α, the root mean squared error (rmse), and the degree-of-freedom adjusted coefficient of determination (R^2) being indicated in the figure legends.

B Noise in the Excitatory Pathway

In the locust visual system, noise increases along the excitatory pathway from the photoreceptors to the LGMD [4]. But to the best of our knowledge there are no data available about the noise levels in the inhibitory pathway. The results of the simulations shown in this study, however, depend critically on noise in the inhibitory pathway. This leads to the question about the impact of excitatory noise on our results. We verified that excitatory noise will not interfere strongly with the predictions of n-ψ. In order to illustrate, we replace Equation (10) by

$$g_{exc}(t) = \frac{\gamma_e}{N_e} \sum_{i=1}^{N_e} \left[\vartheta(t) + \sigma_{\text{exc}} \cdot \xi_i - \Delta_e\right]^+ \quad , \tag{B1}$$

where the subscript "e" denotes the corresponding noise parameters for the excitatory pathway. Supplementary Figure A1 shows that n-ψ peaks slightly later in the presence of excitatory and inhibitory noise, in contrast to having only inhibitory noise. In addition, the n-ψ response gets slightly distorted when having noise in the excitatory pathway.

One important signature of the LGMD is the linear dependence of $t_{\text{rel}} \equiv t_c - t_{\text{max}}$ on l/v [5] (Eq. 2). Supplementary Figure A2a shows that the linear relationship remains intact in the presence of excitatory noise. In addition, the line fit slopes α vary only weakly as a function of the excitatory noise level σ_e, compared with similar variations of the inhibitory noise level (Fig. 4b). Supplementary Figure A2b repeats the simulation from Supplementary Figure A2a with a bigger

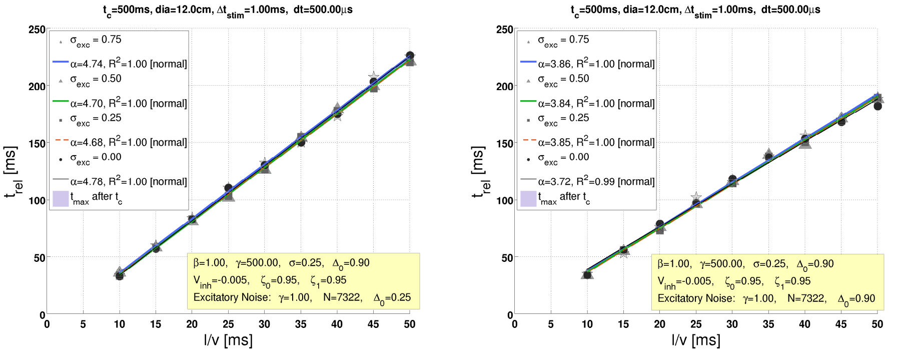

(a) $\Delta_e = 0.25$ **(b)** $\Delta_e = 0.9$

Supplementary Figure A2 | Excitatory noise does not compromise the linear dependence of $t_{\mathbf{rel}}$ from l/v. In both figures, the inhibitory noise level was left constant at $\sigma = 0.25$, and the excitatory noise level σ_e varied. (**a**) Different symbols for data points t_{rel} versus l/v represent different values of excitatory noise σ_e (see figure legend). The continuous lines represent corresponding line fits (linear regression) to data points with the same σ_e. The line slopes α are indicated in the figure legend (where "normal" means that the residuals are normal-distributed). The α do not depend strongly on σ_e. (**b**) Setting a different value for the threshold $\Delta_e = 0.9$ does not compromise the linearity either, but causes only a decrease of the overall slopes α relative to those with $\Delta_e = 0.25$.

threshold Δ_e. This causes an overall decrease of the α, but does not affect linearity.

If the equilibrium solution Equation (7) is used instead of explicitly integrating the differential Equation (6), then the linear dependence of t_{rel} on l/v starts to deteriorate for either $\Delta_e > 0.6$ (with fixed $\sigma_e = 0.5$) or $\sigma_e > 1.6$ (with fixed $\Delta_e = 0.25$).

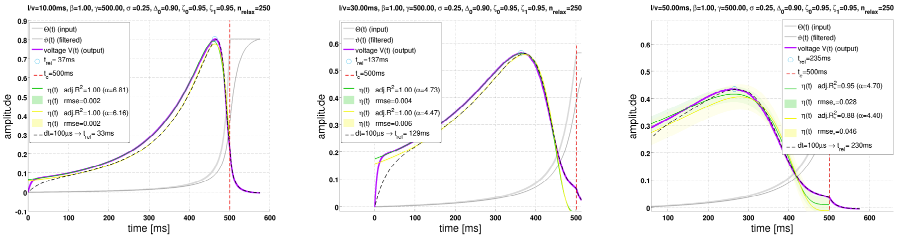

(a) $l/v = 10$ms **(b)** $l/v = 30$ms **(c)** $l/v = 50$ms

Supplementary Figure B1 | Integration time constant $dt = 500\mu s$ versus $dt = 100\mu s$. The figure shows the membrane voltage (=n-ψ-response; Eq. 6) which is integrated with the default integration step size $dt = 500\mu$s (solid violet curve) and with $dt = 100\mu$s (dashed curve). Corresponding fits of the η-function are shown with green and yellow curves, respectively. α, the root mean squared error (rmse), and the degree-of-freedom adjusted coefficient of determination (R^2) of the fitted η-function are indicated in the legends. Collision time is indicated by a vertical dashed red line in each plot. The integration method was Runge-Kutta 4th order. The figure demonstrates that bigger values of dt move the response curves closer to the equilibrium solution V_∞ (see next figure) and thus the curves peak earlier. Each figure panel shows a different halfsize-to-velocity ratio l/v.

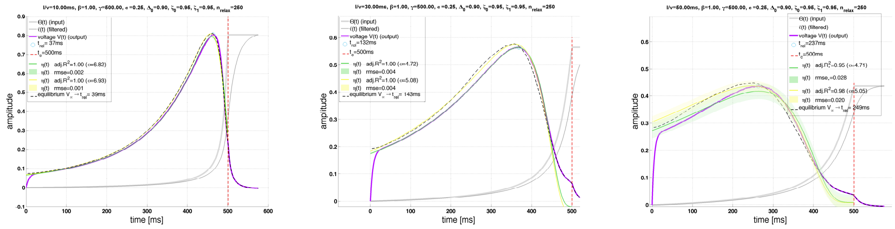

(a) $l/v = 10$ms **(b)** $l/v = 30$ms **(c)** $l/v = 50$ms

Supplementary Figure B2 | Integration with $dt = 500\mu s$ versus steady-state V_∞. This figure compares the n-ψ-responses (violet curve: Eq. 6 integrated with $dt = 500\mu s$) for different halfsize-to-velocity ratios l/v with the equilibrium solution V_∞ (dashed curve: Eq. 7). The fit of the η-function to the equilibrium n-ψ-responses is represented by a yellow curve. The green curve is the fit to the numerically integrated n-ψ-function (see previous figure for further details). The figure suggests that the default integration step size $dt = 500\mu s$ with $n_{relax} = 250$ relaxation steps is very close to the equilibrium solution. Collision time is indicated by a vertical dashed red line in each plot.

C Integration time constant dt and n_{relax}

After each integration step of the membrane potential (=n-ψ -response; Eq. 6) with integration step size dt, a number of relaxation time steps n_{relax} is intercalated. During this "relaxation phase", the excitatory and inhibitory synaptic input (Eqs. 10 and 11, respectively) are held constant. If a suitably small inte-

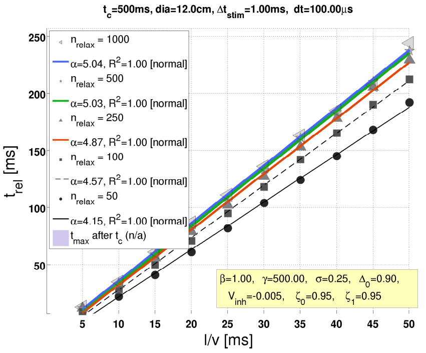

(a) $dt = 100\mu s$

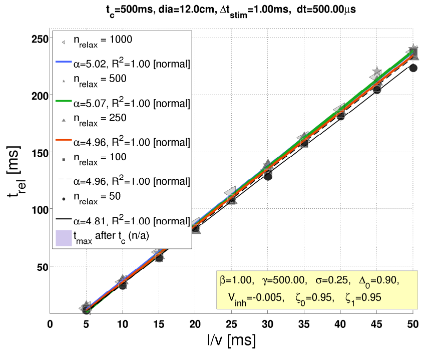

(b) $dt = 500\mu s$

Supplementary Figure B3 | Influence of relaxation steps n_{relax} on the linear dependence of t_{rel} from l/v. Different symbols correspond to different values of n_{relax} as indicated in the figure legends. The continuous lines represent corresponding linear regressions to the data points. The resulting line slopes α are indicated in the legends as well ("normal" means that the residuals are normal-distributed). (**a**) With an integration step size of $dt = 100\mu s$, the number of relaxation time steps n_{relax} have a notable influence on the line slopes, because the n-ψ model is far from its equilibrium solution. Taking more relaxation time steps will move the response maxima of n-ψ away from t_c (i.e., to earlier times), thus t_{rel} increases. (**b**) With the default integration step size $dt = 500\mu s$, n-ψ is already close to its steady-state, and therefore the slope values α depend only weakly on n_{relax}.

gration time constant dt is chosen, it is therefore possible to study the responses of n-ψ close or far from the equilibrium state. In reference [1], stimulation far away from the equilibrium state has been put forward as an explanation of why the response peak of the LGMD neuron could occur *after* collision time t_c. Here we do not repeat those numerical experiments, but are rather interested to select dt (and n_{relax}) such that we are reasonably close to the equilibrium. In Supplementary Figure B1 the n-ψ-responses ($n_{relax} = 250$) with $dt = 100\mu s$ (i.e., somewhat away from equilibrium) are compared with $dt = 500\mu s$ (close to equilibrium). Supplementary Figure B2 compares the n-ψ-responses (default: $dt = 500\mu s$ and $n_{relax} = 250$) with the steady-state solution (V_∞), and confirms that the default values are close to the equilibrium state. Supplementary Figure B3 illustrates the influence of n_{relax} on the maxima of n-ψ -responses for $dt = 100\mu s$ and $dt = 500\mu s$, respectively, and confirms once more that the latter (default) value is close to the equilibrium state.

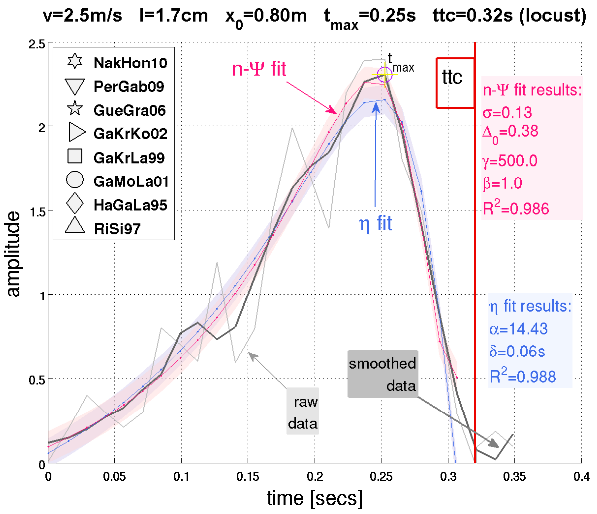

Supplementary Figure C1 | Fitting explained. This figure introduces the symbols and curves that are used for presenting the fitting results. The light gray curve are the raw data from a respective publication. The publication is either denoted by a symbol (Supp. Figs D2 & D3), or a corresponding label (Figure 6a; Supp. Figs D5 to D23), see figure key. In the summary plots (Supp. Figs D2 & D3), data from the same paper that have the same graphical symbol are further distinguished by their respective halfsize to velocity ratio l/v. Only in a few occasions we have ambiguities (i.e. two data sets from one paper have equal l/v ratios). In the individual fits (Figure 6a; Supp. Figs D5 to D23),if a light gray curve is shown at the same time with a dark gray curve, then the latter curve corresponds to smoothed raw data. Otherwise, the dark gray curve are the raw data. The vertical red line indicates the projected time of collision. The fitting parameters for n-ψ are in pink fonts, and those of the η-function in blue fonts. Here, "fit 2 raw" means that n-ψ was fitted to the raw data, and η was fitted to the smoothed data ("fit 2 smooth"). The references for the labels: *NakHon10*=[6], *PerGab09*=[7], *GueGra06*=[8], *GaKrKo02*=[9], *GaKrLa99*=[5], *GaMoLa01*=[10], *HaGaLa95*=[11], and *RiSi97*=[12].

D Fitting the n-ψ and the η-Function to Neuronal Recordings

Collision-sensitive neurons with similar response properties are found in the brains of many mammals and insects [13]. A particular class of these neurons reveal a response maximum when an object approaches the eye with constant velocity. The η-function was the first model for describing the response properties of these neurons [11]. It is commonly used for fitting corresponding neuronal recording curves (of "η-type" neurons). The purpose of this section is to juxtapose fitting results of the η-function with those of n-ψ. To this end, 36 data sets from eight publications were scanned and fitted (Supplementary Fig. C1a), analogous to Supplementary Section S4 in reference [13]. Thus, many previously

label(s)	fitted function	fitting parameters
TR3, LM3	η	$\{A, \alpha, o\}$
TR4, TR41	η	$\{A, \alpha, \delta, o\}$
TR2	n-ψ	$\{A, o\}$
TR32, TR40	n-ψ	$\{A, \beta, \gamma, \sigma, o\}$
TR444	n-ψ (Fig. 7a)	$\{\sigma, \Delta_0\}$
TR44	n-ψ (Fig. 7b)	$\{\beta, \sigma, \Delta_0\}$
TR50	n-ψ	$\{A, \beta, \gamma, \sigma, \Delta_0, o\}$

Table D1 | Labels and fitting parameters The table relates the set of fitted parameters to the corresponding labels as they are indicated in the legends. *TR* means that the Trust region algorithm was used for fitting [14, 15]. *LM* means that the Levenberg-Marquardt algorithm was used [16])

published data sets are united with a common fitting framework, what allows for an unequivocal and unbiased evaluation of the fitted functions. The data sets covered halfsize-to-velocity ratios from l/v = 5ms to 50ms.

D.1 Details for Fitting the η-Function

The fitting model for the η-function was (cf. Eq. 1)

$$A\dot{\Theta}(t+\delta)e^{-\alpha\Theta(t+\delta)} + o \tag{D1}$$

with A = amplitude, δ = temporal delay, o = offset, = Θ angular size, $\dot{\Theta}$ = angular velocity, and α is a weight. For each experimentally obtained response curve from the LGMD, two parameter sets combined with two fitting algorithms were tested. The best result in terms of goodness of fit measures was then selected from the four combinations. For Supplementary Equation (D1), the parameter set to be determined was either $\{A, \delta, o, \alpha\}$ or $\{A, o, \alpha\}$ (with $\delta = 0$). The fitting method was either Trust-Region (legend label "*TR*", [14, 15]), or Levenberg-Marquardt ("*LM*", [16]). The respective fitting procedures are indicated in the figure legends by corresponding labels which are listed in Table D1.

D.2 Details for Fitting the ψ-Model

For the purpose of comparison, fitting results for the ψ-model (see reference [1]) are shown in Supplementary Figure D2. The fitting model for ψ is based on Equation (7):

$$A \cdot V_\infty(t) + o \tag{D2}$$

with A = amplitude and o = offset. The ψ-model is defined by assigning excitatory and inhibitory input, respectively, in Equation (7) as follows:

$$g_{exc}(t) = \dot{\Theta}(t) \tag{D3a}$$

$$g_{inh}(t) = [\gamma\Theta(t)]^e \tag{D3b}$$

where γ = synaptic weight and e is an exponent (notice that ψ was fit without temporal delay). Both the Trust-Region algorithm [14, 15] and the Levenberg-Marquardt algorithm were used [16].

D.3 Details for Fitting the n-ψ-Model

For fitting n-ψ, only the Trust-Region algorithm was used [14, 15]. The fitting model for n-ψ is based on Equation (7):

$$A \cdot V_{\infty}(t) + o \tag{D4}$$

with A = amplitude and o = offset. No lowpass filtering was employed for fitting, thus $\zeta_0 = \zeta_1 = 0$ in Equations (8) and (9), respectively. The n-ψ-model is defined by assigning excitatory and inhibitory input, respectively, according to Equations (10) and (11). However, Equation (11) cannot be used readily with a conventional fitting algorithm, because of thresholding. For this reason, we used the explicit solution instead, which is smooth (Supplementary Eq. A7: $V \equiv g_{inh}$ and $x \equiv \vartheta$). The legend labels in the fitting results relate to the fitted parameters as shown in Table D1. For example, "*TR50*" means that parameters $\{A, \beta, \gamma, \sigma, \Delta_0, o\}$ were determined by the Trust-Region algorithm, and "*TR40*" refers to the same set of parameters but excluding Δ_0. In the latter case, Δ_0 was held constant at 0.9.

Why fitting the noise level σ and not keeping it constant instead? The underlying idea is to use the n-ψ-model to measure the noise level directly from the different data sets. The noise level ideally should settle around some value then. However, different factors such as body temperature or stimulation conditions (light level, stimulus velocity) could influence the effective noise level across different studies [4, 13], so some spread could be expected around the average value of σ.

D.4 Summary Results

We fitted the η-function, the ψ-model, and the n-ψ-model to the data sets referenced by Supplementary Figure C1. We then plotted the 'important" fitting parameters of these models versus l/v ("important" are those model parameters which determine the location of the activity maximum). Supplementary Figure D2a shows the dependence of α of the η-function from l/v. The median value $\pm 1\sigma_{rob}$ is 3.1 ± 0.7, where the α were more scattered for smaller l/v.

Supplementary Figure D2b shows the exponent e of the ψ-model versus l/v, where $e = 2.7 \pm 0.5$ (median $\pm 1\sigma_{rob}$). Although most of the values fall approximately between 2 and 3.5, occasionally much bigger values were obtained across the whole l/v domain.

For the n-ψ -model, we get the median noise level $\sigma = 0.4 \pm 0.1$ (Supplementary Fig. D3a), and the median threshold $\Delta_0 = 0.9 \pm 0.1$ (Supplementary Fig. D3b). Note, however, that Δ_0 was held constant 0.9 while fitting as far as possible. If Δ_0 was to be determined by the fitting algorithm, then these values are often different from 0.9. This variation in Δ_0 is reflected also in the σ: The highest deviations of σ from its median value are observed when Δ_0 was fitted at the same time.

We asked whether some parameters of the different models correlated across the fitted data. Correlations between two parameters could be suggestive of that they play similar roles in the considered models.

Supplementary Figure D4 visualizes the correlations between most variables. In order to achieve a better visualization, positive correlations are displayed above the diagonal (=self-correlations), and negative values below (cf. figure

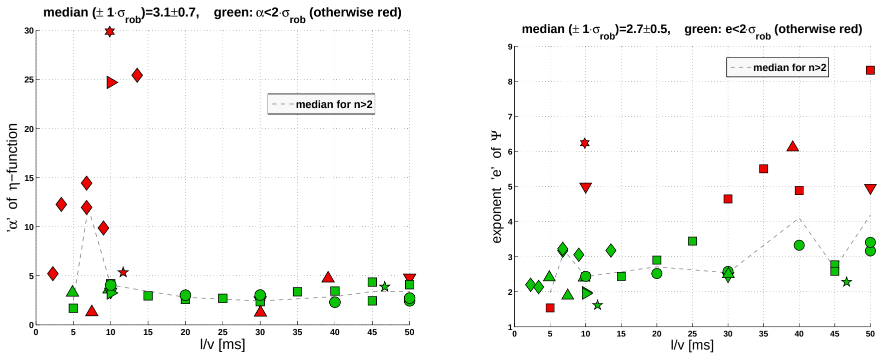

(a) η-function: α **(b)** ψ-function: exponent $\mathbf{e}$

Supplementary Figure D2 | Fit results I The figure shows the fitting results of the important model parameters of η and ψ, respectively, to the neuronal data sets which can be identified by their symbols (cf. Fig. C1): (**a**) α versus l/v (η-function fit, Eq. D1). (**b**) The exponent e versus l/v (ψ-function fit, Eq. D3b). The broken lines denote the median value if more than one value of α (or e) was available at some l/v. Green symbols mark values which were smaller than two times the robust estimation of spread (”σ_{rob}”) via the median absolute deviation (”MAD”), that is $\sigma_{rob} \equiv$ MAD/0.6745 (e.g. [17]). For both function, the green symbols appear to be reasonably independent from l/v. Since the α and e, respectively, determine the location of the LGMD's response maximum, their approximate independence from l/v may indicate a specific tuning of the LGMD to certain ecological conditions. In agreement with the latter interpretation, the bullfrog-data (six-pointed stars; [6]) seem to suggest much bigger values – but this has to be confirmed with a bigger sample size.

legend for more details). No correlation value was sufficiently high in order to suggest a possible link between any parameter pair. The highest correlation was -0.51 between e and σ, and the correlation between σ and Δ_0 was 0.77. The relative high correlation between σ and Δ_0 can be explained by the fitting procedure, where strong co-variations can be observed between both values in Supplementary Figure D3 if Δ_0 and σ were fitted together (compared to holding Δ_0 constant at 0.9). Furthermore, it is likely that the low correlation values are a consequence of our sample size being too small (36 data sets), because statistical variations in the fitting results did not average out.

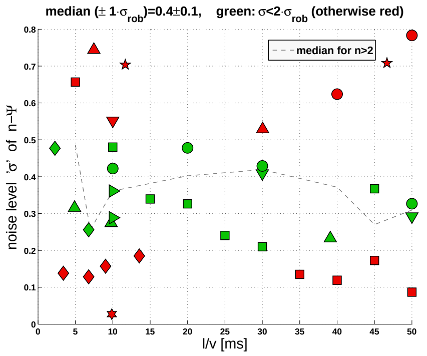

(a) n-ψ-function: σ

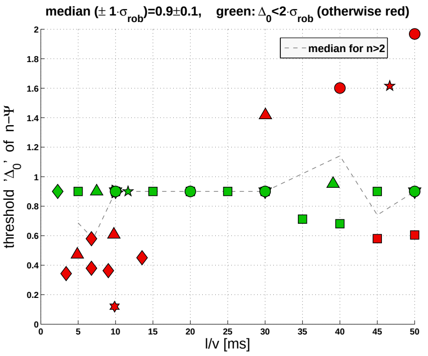

(b) n-ψ-function: $\boldsymbol{\Delta_0}$

Supplementary Figure D3 | Fit results II The figures show fitting results of two n-ψ-parameters: The noise level σ and the threshold Δ_0. σ was fitted rather than being set to some fixed value in order to "measure" noise levels from the the neuronal data sets (identifiable by their symbols, see Fig. C1). Δ_0 was only fitted if goodness of fit measures with a fixed Δ_0 were poor. (**a**) Noise level σ versus l/v (n-ψ-function fit, Eq. 11). The median noise level (here dimensionless) is $\sigma = 0.4 \pm 0.1$ (mean $\pm$ 1 SD), what should be compared with the experimentally found values from ref. [4] (p. 1071: mean spontaneous noise levels relative to rest in photoreceptors = 0.19mV, LMCs = 0.4mV, and LGMD=1.05mV). (**b**) Threshold Δ_0 versus l/v (n-ψ-function fit, Eq. 11). The broken lines denote the median value if more than one value of σ (or Δ_0) was available at some l/v. Green symbols mark values which were smaller than two times the robust estimation of spread ("σ_{rob}") via the median absolute deviation ("MAD"), that is $\sigma_{rob} \equiv \mathrm{MAD}/0.6745$ (e.g. [17]).

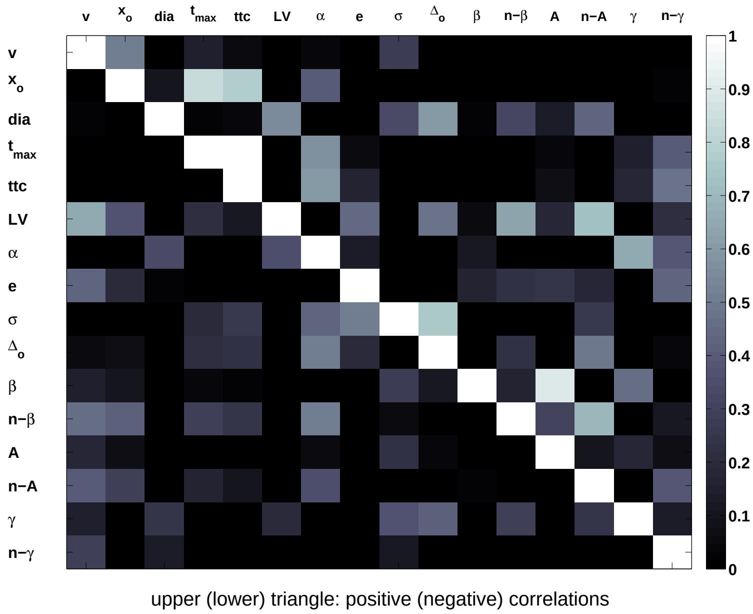

Supplementary Figure D4 | Fit results III: Correlations This figure displays the correlations between various model parameters. Positive correlations are above the diagonal (= white squares), and negative correlations are shown below. Labels: LV $= l/v$, diameter dia $= 2l$; β, A and γ refer to the ψ-model, while n-β, n-A and n-γ refer to n-ψ. The highest correlation (0.9) occurs between β and A of the ψ model. For n-ψ, the correlation between n-β and n-A is 0.7, the correlation between σ and Δ_0 is 0.77, and between n-A and LV it is 0.72. Furthermore, across the "important" model parameters, we found -0.51 between e and σ, -0.42 between α and σ, and finally 0.13 between α and e.

D.5 Detailed Results

(This section is analogous to Supplementary Text S4 in reference [13]). The Supplementary Figures D5 to D23 juxtapose the detailed fitting results of the η-function with those of n-ψ . Each figure legend has a label that identifies the data set via Figure Supplementary C1. Goodness of fit measures and model parameters are indicated as well.

The target data for fitting are always plotted with a thick and dark gray line. Occasionally the noisy neuronal data were smoothed. In that case, the original neuronal data are drawn with a thin and light gray line, and the smoothing method (along with its corresponding parameter value) is indicated as follows:

"*robLR1st*" robust local regression with weighted linear least squares and a *1st degree* polynomial model. The parameter specifies the span (= number of data points for computing a smoothed value) in terms of percentage of total number of data points.

"*robLR2nd*" robust local regression with weighted linear least squares and a *2nd degree* polynomial model. The parameter specifies the span in terms of percentage of total number of data points.

"*sgolay*" Savitzky-Golay method with polynomial degree 2. Parameter value p means that the span in terms of number of data points is $2p + 1$.

If no clear response peak could be detected in the neuronal data, or if successive data points were too separated in time, we proceeded with selecting a smoothing algorithm. In that case, the tuning criteria were *(i)* to leave the original data as less distorted as possible, and *(ii)* to assure, by visual inspection, a sound location of the response peak in the smoothed data. This is to say that $t_{\max}$ of the smooth data should coincide with where an experienced observer would place it.

The label "*fit 2 smooth*" means that η or n-ψ were fit to the smoothed data, and "*fit 2 raw*" means that the corresponding models was fitted to the non-smoothed (= raw) data.

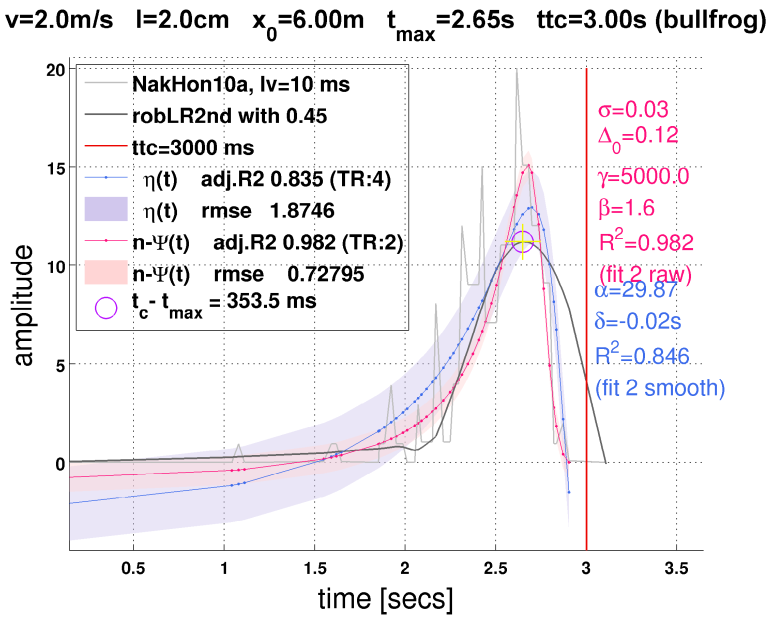

Supplementary Figure D5 | NakHon10 Bullfrog *rana catesbeiana*, $l/v =$ 10ms, figure 8 and 9, respectively, from reference [6]. The stimulus was a 35mm × 35mm black square ($l \approx$ 2cm), $t_{\max}$ at around 2600ms (400ms before t_c). (Abscissa in units of seconds). Table D1 relates the legend labels to the set of fitted parameters.

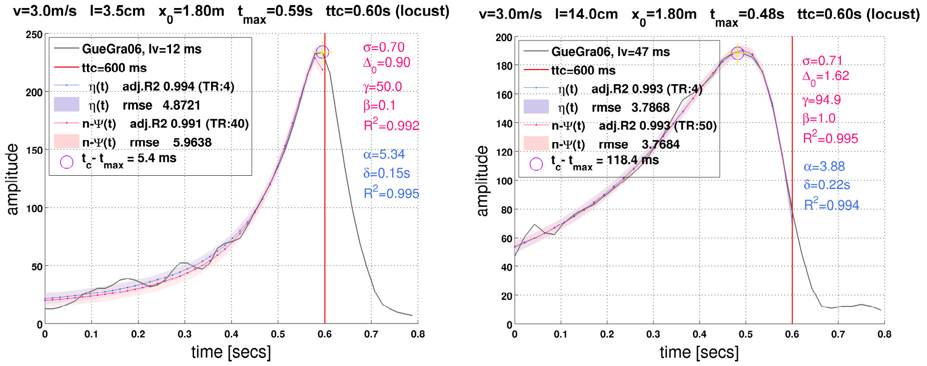

(a) $l/v = 11.7$ms **(b)** $l/v = 46.7$ms

Supplementary Figure D6 | GueGra06 Locust. Figure from reference [8]. The stimulus was a black disk. (Abscissa in units of seconds). Table D1 relates the legend labels to the set of fitted parameters.

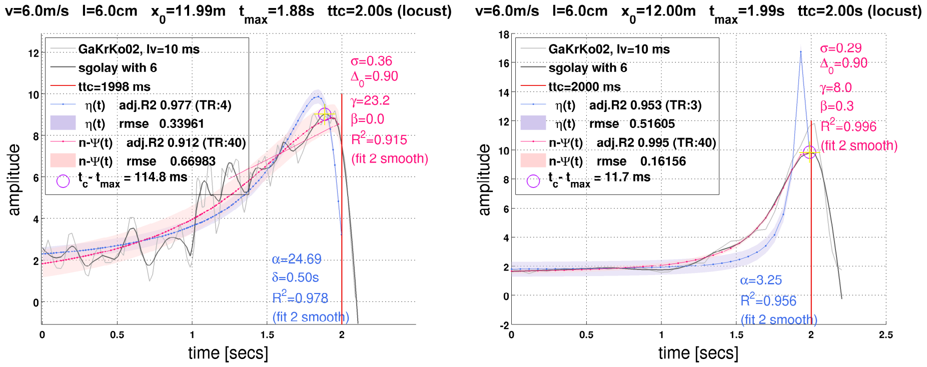

(a) control **(b)** TTX-injection

Supplementary Figure D7 | GaKrKo02 Locust. Figure 4b from reference [9], $l/v = 10$ms, black squares. (Abscissa in units of seconds). Table D1 relates the legend labels to the set of fitted parameters.

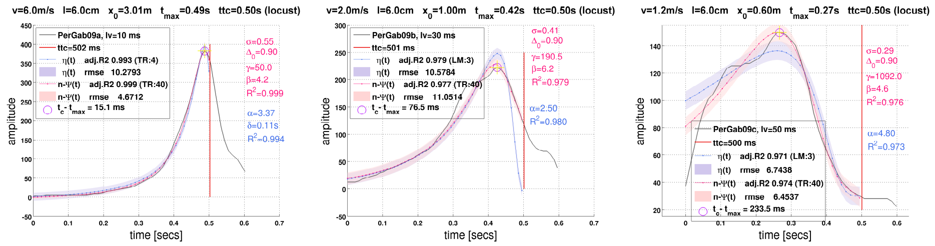

(a) $l/v = 10$ms **(b)** $l/v = 30$ms **(c)** $l/v = 50$ms

Supplementary Figure D8 | PerGab09 Locust. Figure 4 from reference [7], black disks. (Abscissa in units of seconds). Table D1 relates the legend labels to the set of fitted parameters.

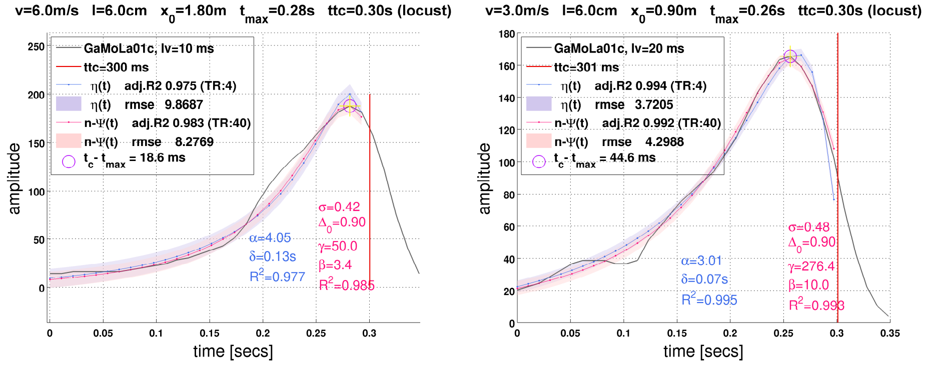

(a) $l/v = 10$ms **(b)** $l/v = 20$ms

Supplementary Figure D9 | GaMoLa01 I Locust. Figure 1c from reference [10], looming squares. (Abscissa in units of seconds). Table D1 relates the legend labels to the set of fitted parameters.

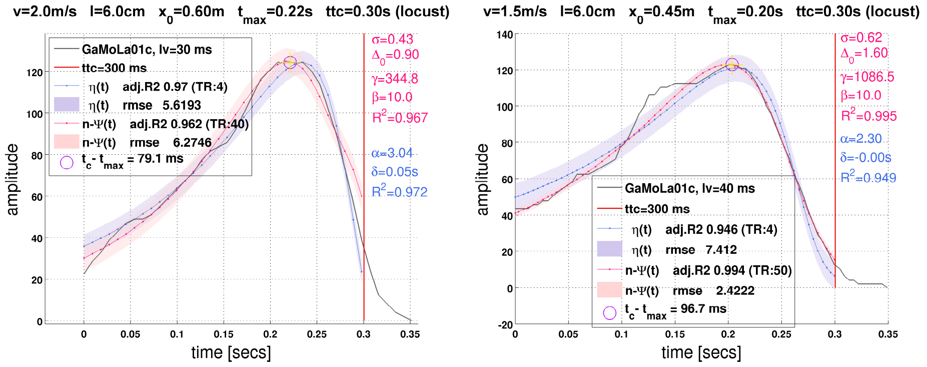

(a) $l/v = 30\text{ms}$ **(b)** $l/v = 40\text{ms}$

Supplementary Figure D10 | GaMoLa01 II Locust. Figure 1c from reference [10], looming squares. (Abscissa in units of seconds). Table D1 relates the legend labels to the set of fitted parameters.

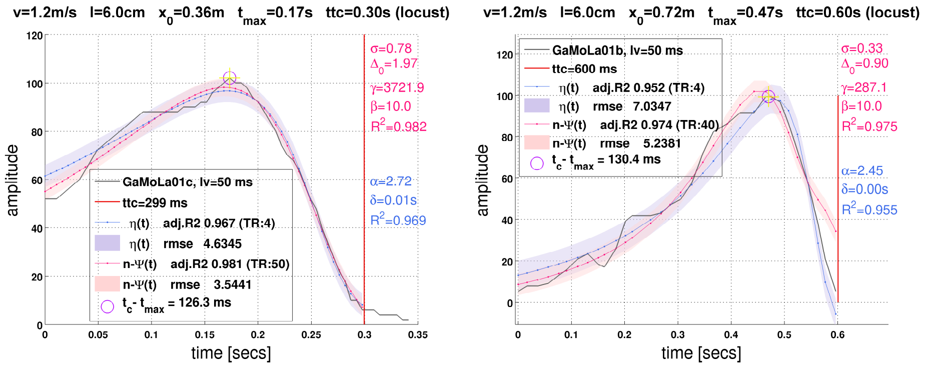

(a) $l/v = 50\text{ms}$ **(b)** $l/v = 50\text{ms}$

Supplementary Figure D11 | GaMoLa01 III Locust. (**a**) Figure 1c, (**b**) Figure 1b, both from reference [10], looming squares. (Abscissa in units of seconds). Table D1 relates the legend labels to the set of fitted parameters.

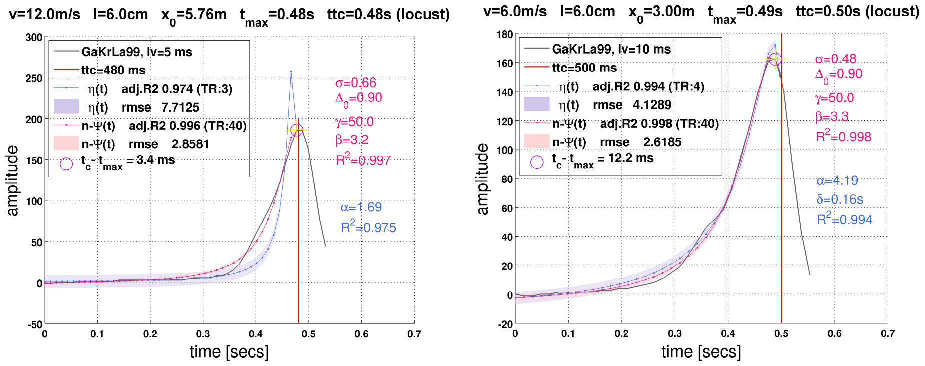

(a) $l/v = 05\text{ms}$ **(b)** $l/v = 10\text{ms}$

Supplementary Figure D12 | GaKrLa99 I Locust. Figure 3 from reference [5], dark squares. (Abscissa in units of seconds). Table D1 relates the legend labels to the set of fitted parameters.

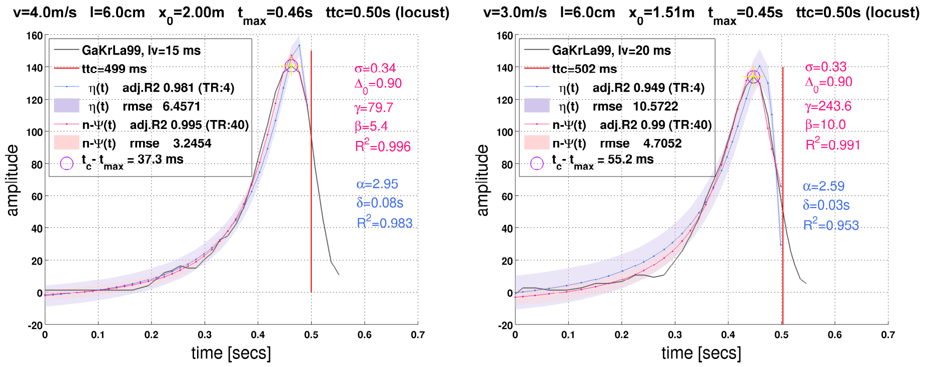

(a) $l/v = 15\text{ms}$ **(b)** $l/v = 20\text{ms}$

Supplementary Figure D13 | GaKrLa99 II Locust. Figure 3 from reference [5], dark squares. (Abscissa in units of seconds). Table D1 relates the legend labels to the set of fitted parameters.

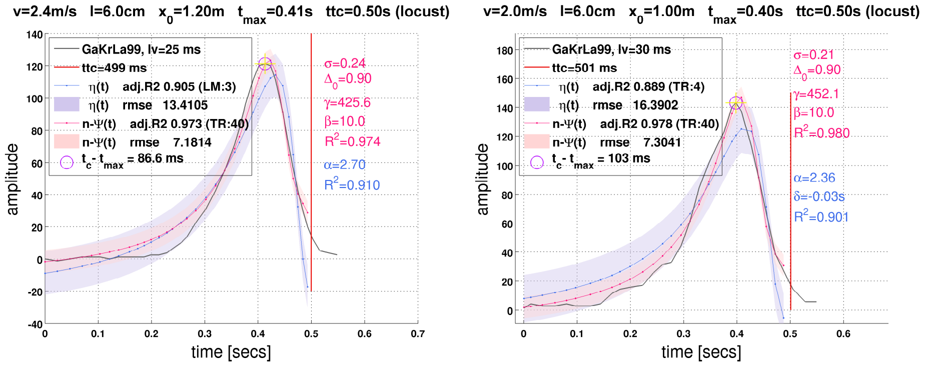

(a) $l/v = 25\text{ms}$ **(b)** $l/v = 30\text{ms}$

Supplementary Figure D14 | GaKrLa99 III Locust. Figure 3 from reference [5], dark squares. (Abscissa in units of seconds). Table D1 relates the legend labels to the set of fitted parameters.

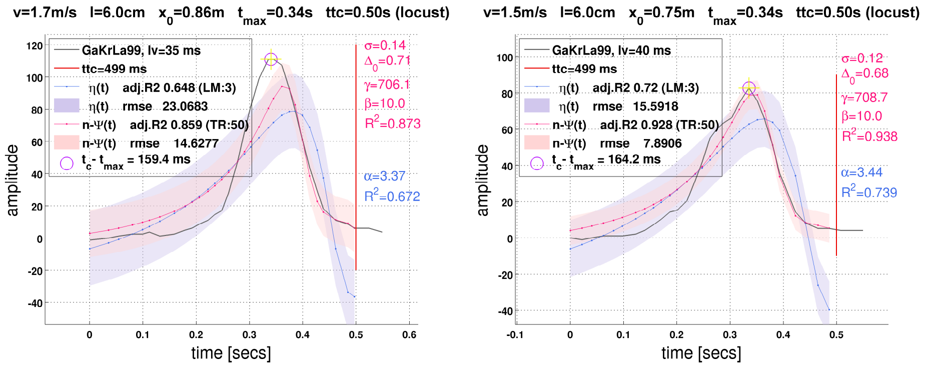

(a) $l/v = 35\text{ms}$ **(b)** $l/v = 40\text{ms}$

Supplementary Figure D15 | GaKrLa99 IV Locust. Figure 3 from reference [5], dark squares. (Abscissa in units of seconds). Table D1 relates the legend labels to the set of fitted parameters.

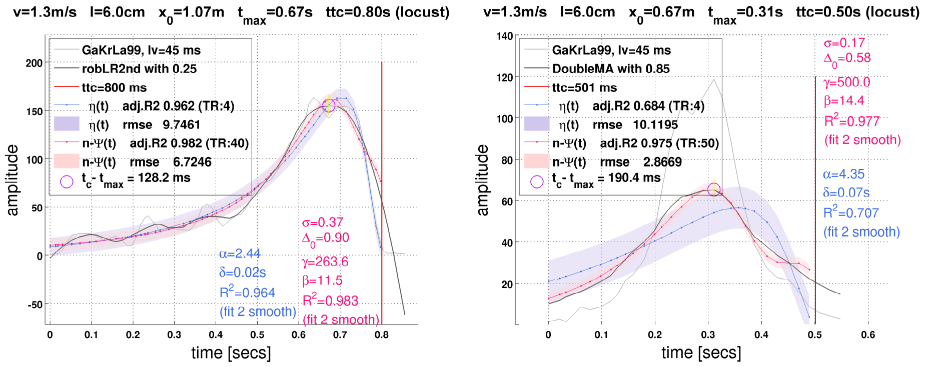

(a) $l/v = 45\text{ms}$ **(b)** $l/v = 45\text{ms}$

Supplementary Figure D16 | GaKrLa99 V Locust. (**a**) Figure 2, (**b**) Figure 3, both from reference [5], dark squares. (Abscissa in units of seconds). Table D1 relates the legend labels to the set of fitted parameters.

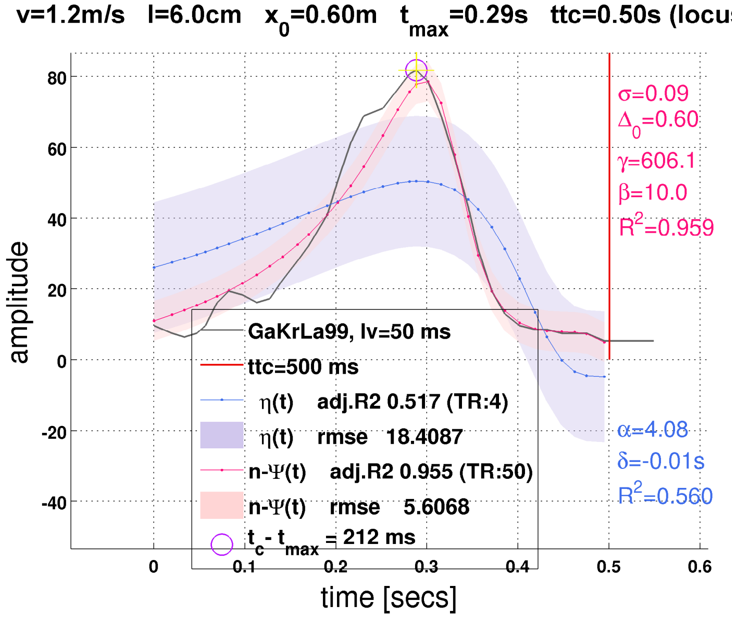

Supplementary Figure D17 | GaKrLa99 VI Locust, $l/v = 50\text{ms}$. Figure 3 from reference [5], dark squares. (Abscissa in units of seconds). Table D1 relates the legend labels to the set of fitted parameters.

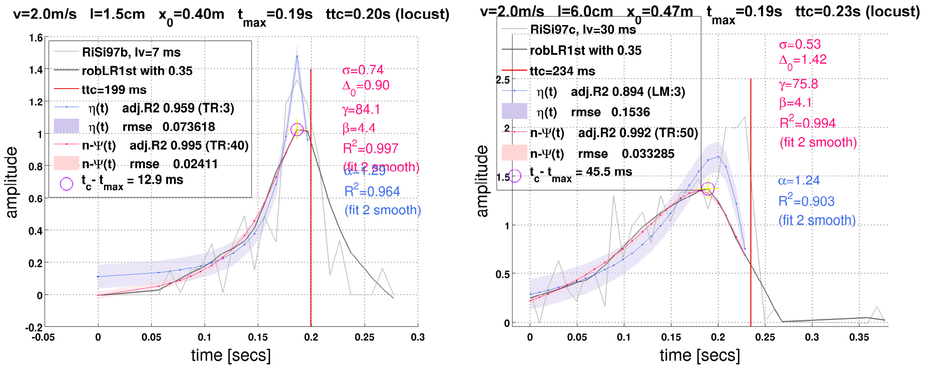

(a) $l/v = 7\text{ms}$ **(b)** $l/v = 30\text{ms}$

Supplementary Figure D18 | RiSi97 I Locust, Figure 1B, C from reference [12], approach speed $v = 2\text{m/s}$, (**a**) disk 17° ($l \approx 0.015m$) (**b**) disk 62° ($l \approx 0.06m$). (Abscissa in units of seconds). Table D1 relates the legend labels to the set of fitted parameters.

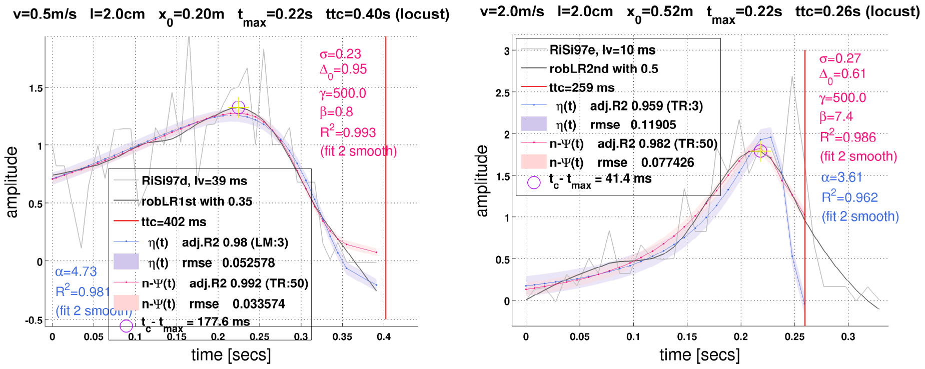

(a) $l/v = 39\text{ms}$ **(b)** $l/v = 10\text{ms}$

Supplementary Figure D19 | RiSi97 II Locust, Figure 1D, E from reference [12], 30mm × 40mm black rectangle ($l \approx 0.02m$) approach speed (**a**) $v = 0.5\text{m/s}$, (**b**) $v = 2\text{m/s}$. (Abscissa in units of seconds). Table D1 relates the legend labels to the set of fitted parameters.

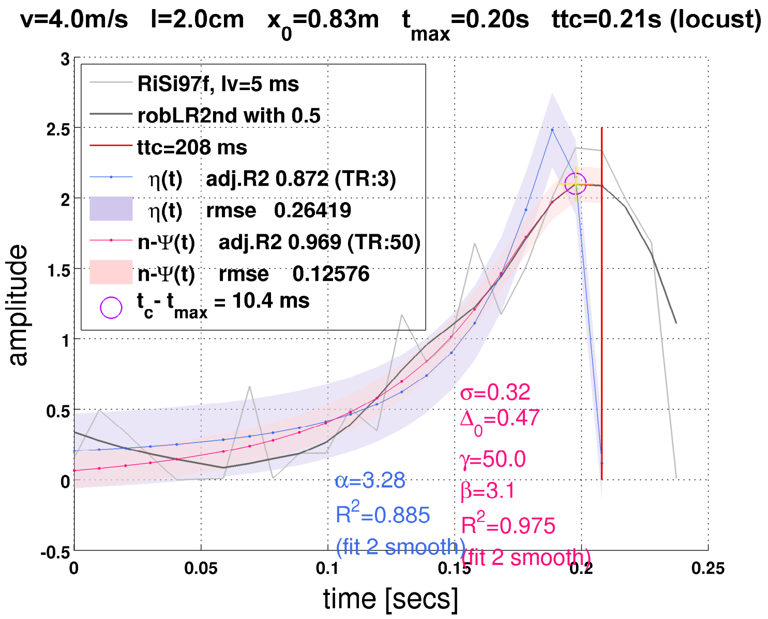

Supplementary Figure D20 | RiSi97 III Locust, $l/v = 5$ms. Figure 1F from reference [12], 30mm × 40mm black rectangle ($l \approx 0.02m$, approach speed $v = 4$m/s). (Abscissa in units of seconds). Table D1 relates the legend labels to the set of fitted parameters.

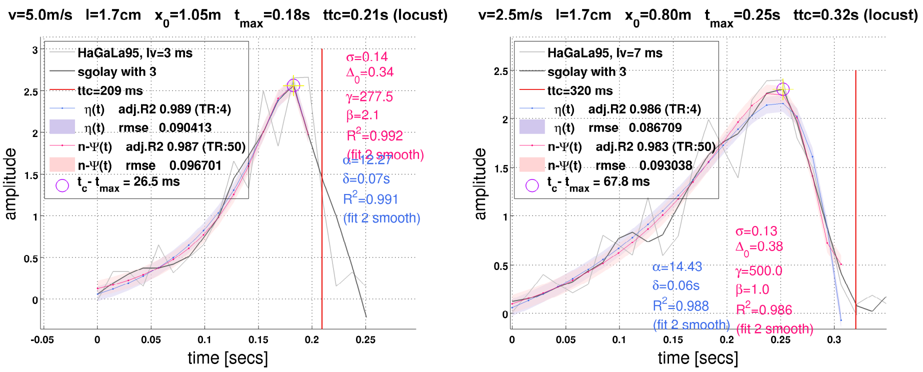

(a) $l/v = 3.39$ms **(b)** $l/v = 6.77$ms

Supplementary Figure D21 | HaGaLa95 I Locust, Figure 3Ai, Aii from reference [11], 3cm black square ($l \approx 0.017m$) approach speed (**a**) $v = 5$m/s, (**b**) $v = 2.5$m/s. (Abscissa in units of seconds). Table D1 relates the legend labels to the set of fitted parameters.

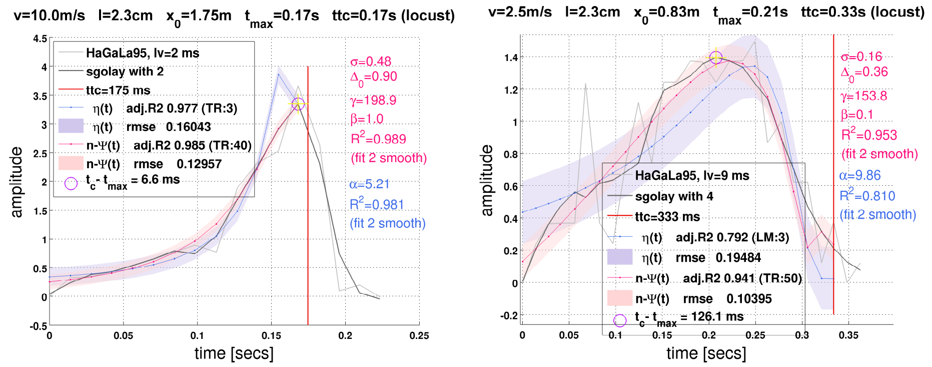

(a) $l/v = 2.26$ms **(b)** $l/v = 9$ms

Supplementary Figure D22 | HaGaLa95 II Locust, Figure 3Bi, Bii from reference [11], 4cm black square ($l \approx 0.023m$) approach speed (**a**) $v = 10$m/s, (**b**) $v = 2.5$m/s. (Abscissa in units of seconds). Table D1 relates the legend labels to the set of fitted parameters.

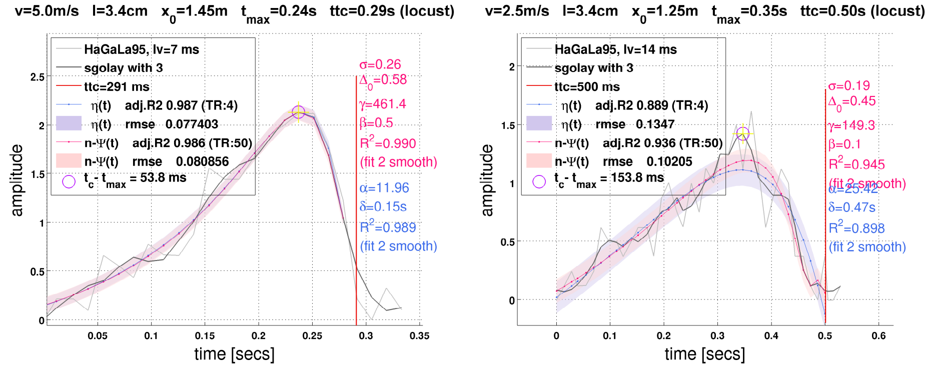

(a) $l/v = 6.77$ms **(b)** $l/v = 13.54$ms

Supplementary Figure D23 | HaGaLa95 III Locust, Figure 3Ci, Cii from reference [11], 6cm black square ($l \approx 0.034m$) approach speed (**a**) $v = 5$m/s, (**b**) $v = 2.5$m/s. (Abscissa in units of seconds). Table D1 relates the legend labels to the set of fitted parameters.

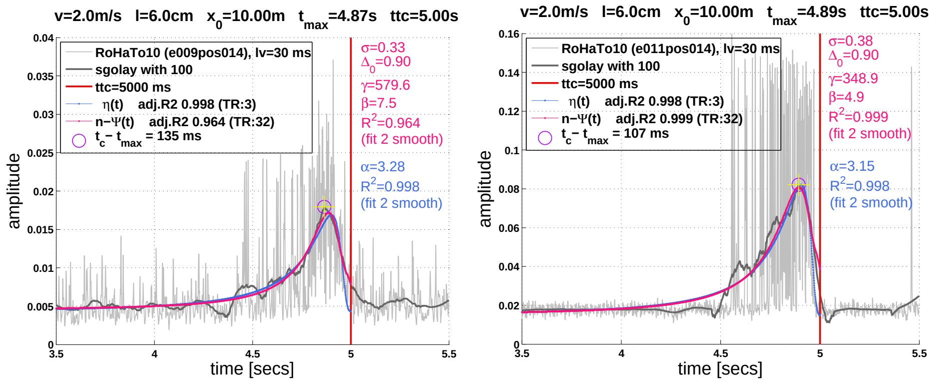

(a) e009pos014 **(b)** e011pos014

Supplementary Figure D24 | (See Figure 6 for details).

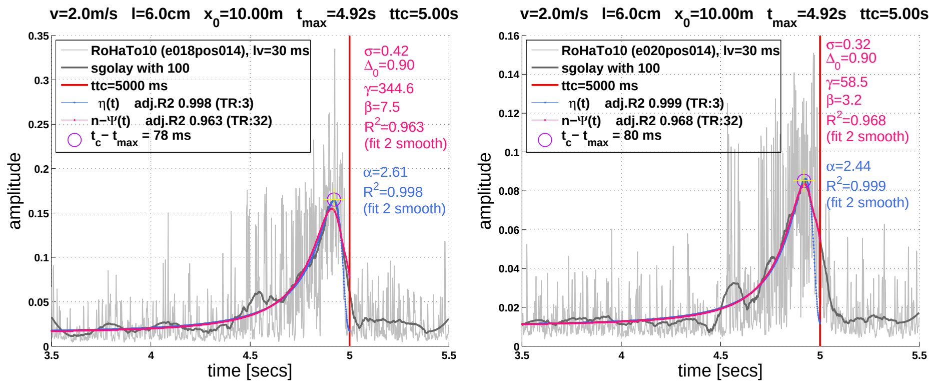

(a) e018pos014 **(b)** e020pos014

Supplementary Figure D25 | (See Figure 6 for details).

D.6 Fit to Recording Traces

Figures 6, D24 and D25 show recording traces from the *Descending Contralateral Movement Detector* neuron (DCMD). All five recording traces were kindly provided by Steve Rogers [18]. The DCMD replicates the firing patterns of the LGMD (cf. [19, 20, 21]). The spike traces (sampled at $10^4 Hz$) were full wave rectified, lowpass filtered, and sub-sampled to 1ms resolution. Firing rates were estimated with Savitzky-Golay filtering ("*sgolay*"). The η-function and n-ψ were fitted to these firing rate estimates with the Trust-Region algorithm [14, 15]. The respective parameter sets were $\{A, \alpha, o\}$ for the η-function ("*TR:3*") and $\{A, \beta, \gamma, \sigma, o\}$ for n-ψ ("*TR:32*"). For all fits, the temporal delay was set to zero ($\delta = 0$).
The results can be summarized in just a few words: Both functions describe the data excellently, and η fits them marginally better than n-ψ.

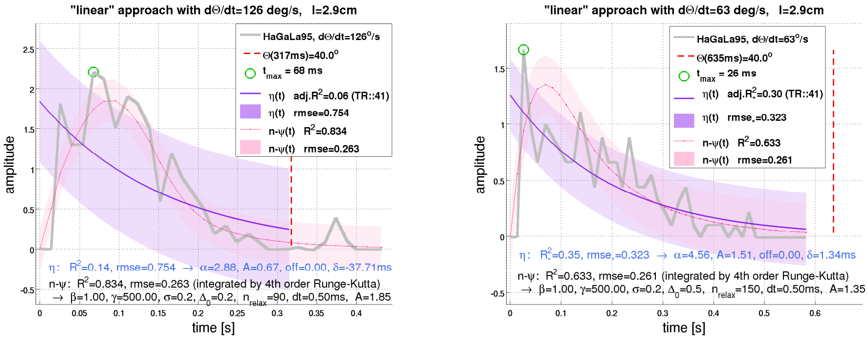

(a) $\dot{\Theta} = 126^o/s$ **(b)** $\dot{\Theta} = 63^o/s$

Supplementary Figure D26 | This figure is analogous to Figure 7 in the main text. The difference is that instead of fitting n-ψ_∞, here the dynamic version of n-ψ (equation 6) was used. This of course implies transient effects, which are reflected at the beginning by an increase in activity to a maximum. For this reason, the (dynamic) n-ψ-predictions were manually adjusted (trial and error) in order achieve a good match with the smoothed version of the recording curve was achieved (the smoothed curves are not shown, though). The optimization criterion was the maximization of the coefficient of determination (R^2). Even in this way n-ψ matches the original (i.e. unsmoothed) data better than the η-function. All goodness of fit measures in this figure refer to the unsmoothed data.

E List of Symbols

symbol	eq.	description
Θ	12	angular size
$\dot{\Theta}$	13	angular velocity or rate of expansion
$\ddot{\Theta}$	4	angular acceleration
t		time (independent variable)
T		delayed time $T = t + \delta$ (used with η)
t_c		time to collision (constant)
v		speed of approaching object (constant)
x_0		initial distance at which the approach starts, $x_0 = v \cdot t_c$ (constant)
$x(t)$		running distance until collision, $x(t) = x_0 - vt$
l		an object's half diameter
l/v		halfsize-to-velocity ratio
$t_{\max}$		time of activity maximum
$T_{\max}$		delayed $t_{\max}$, $T_{\max} = t_{\max} + \delta$
Θ_{max}	14	$\Theta_{max} \equiv \Theta(T_{\max})$, angular size at $t_{\max} + \delta$
t_{rel}		$= t_c - t_{\max}$ (time from peak to t_c)
T_{rel}		$= t_c - T_{\max}$
α	1	inhibitory strength of η-function...
	2	...and at the same time line slope of T_{rel} versus l/v
δ	1	temporal delay between the peak at $t_{\max}$ and angular size $\Theta(t_{\max})$...
	2	...and at the same time line intercept of T_{rel} versus l/v
$g_p(t)$	3	p-th order power law: $g_p(t) \equiv [\log(\eta)]^p$
β	6	leakage conductance (n-ψ-model)
C_m	6	membrane capacitance (set to unity)
τ_m		membrane time constant $\tau_m \equiv C_m/\beta$
V	6	membrane voltage and output of n-ψ
V_∞	7	steady-state membrane voltage
V_{rest}	6	resting potential(n-ψ-model)
g_{exc}	6	excitatory input to n-ψ where $g_{exc} \geq 0$
g_{inh}	6	inhibitory input to n-ψ where $g_{inh} \geq 0$
V_{exc}	6	excitatory reversal potential; upper bound to V: $V \leq V_{exc}$
V_{inh}	6	inhibitory reversal potential; lower bound to V: $V \geq V_{inh}$
ϑ	8	low-pass filtered angular size
$\dot{\vartheta}$	9	low-pass filtered rate of expansion (angular velocity)
ζ_0	8	filter memory constant: no memory $0 \leq \zeta_0 \leq 1$ infinite memory
ζ_1	9	filter memory constant for lowpass filtering of angular velocity
Δt_{stim}	8,9	stimulation time scale (temporal resolution of Θ & $\dot{\Theta}$)
γ	11	inhibitory synaptic weight (for n-ψ but also for fitting ψ, cf. eq. D3b)
N	11	pool size (number of inhibitory processes to be averaged)
ξ_i	11	Gaussian random variable with mean zero and standard deviation one
σ	11	standard deviation of noise in the inhibitory pathway
Δ_0	11	threshold
n_{relax}		number of relaxation time steps (see Methods and Supp. Section C)
dt		integration time step (see Methods and Supp. Section C)
γ_e	B1	excitatory synaptic weight (excitatory noise, see Supp. Section B)
N_e	B1	pool size (excitatory noise, see Supp. Section B)
σ_{exc}	B1	standard deviation of noise in the excitatory pathway
Δ_e	B1	threshold for excitatory noise
e		power law exponent (used in Figure 3 and in the ψ-model Eq. D3b)
δ	D1	temporal delay for fitting of the η-function
A	D1	amplitude for fitting the η-function, ψ, and n-ψ
o	D1	additive constant ("offset") for fitting the η-function, ψ, and n-ψ

References

1. Keil MS. Emergence of Multiplication in a Biophysical Model of a Wide-Field Visual Neuron for Computing Object Approaches: Dynamics, Peaks, & Fits. In: Shawe-Taylor J, Zemel RS, Bartlett P, Pereira FCN, Weinberger KQ, editors. Advances in Neural Information Processing Systems 24; 2011. p. 469–477. Available from: http://books.nips.cc.
2. Miller KD, Troyer TW. Neural noise can explain expansive, power-law nonlinearities in neuronal response functions. Journal of Neurophysiology. 2002;87:653–659.
3. Hansel D, van Vreeswijk C. How Noise Contributes to Contrast Invariance of Orientation Tuning in Cat Visual Cortex. The Journal of Neuroscience. 2002;22(12):5118–5128.
4. Jones PW, Gabbiani F. Impact of neural noise on a sensory-motor pathway signaling impending collision. Journal of Neurophysiology. 2012;107:1067–1079.
5. Gabbiani F, Krapp HG, Laurent G. Computation of object approach by a wide-field, motion-sensitive neuron. Journal of Neuroscience. 1999;19(3):1122–1141.
6. Nakagawa H, Hongjian K. Collision-Sensitive Neurons in the Optic Tectum of the Bullfrog, Rana catesbeiana. Journal of Neurophysiology. 2010;104(5):2487–2499.
7. Peron S, Gabbiani F. Spike frequency adaptation mediates looming stimulus selectivity. Nature Neuroscience. 2009;12(3):318–326.
8. Guest BB, Gray JR. Responses of a Looming-Sensitive Neuron to Compound and Paired Object Approaches. Journal of Neurophysiology. 2006;95:1428–1441.
9. Gabbiani F, Krapp HG, Koch C, Laurent G. Multiplicative computation in a visual neuron sensitive to looming. Nature. 2002;420:320–324.
10. Gabbiani F, Mo C, Laurent G. Invariance of angular threshold computation in a wide-field looming-sensitive neuron. Journal of Neuroscience. 2001;21(1):314–329.
11. Hatsopoulos N, Gabbiani F, Laurent G. Elementary computation of object approach by a wide-field visual neuron. Science. 1995;270:1000–1003.
12. Rind FC, Simmons PJ. Signaling of Object Approach by the DCMD Neuron of the Locust. Journal of Neurophysiology. 1997;77:1029–1033.
13. Keil MS, López-Moliner J. Unifying Time to Contact Estimation and Collision Avoidance across Species. PLoS Computational Biology. 2012;8(8):e1002625.
14. Moré JJ, Sorensen DC. Computing a trust region step. SIAM Journal on Scientific and Statistical Computing. 1983;4(3):553–572.
15. Byrd RH, Schnabel RB, Shultz GA. A Trust Region Algorithm for Nonlinearly Constrained Optimization. SIAM Journal on Numerical Analysis. 1987;24(5):1152–1170.
16. Press WH, Teukolsky SA, Vetterling WT, Flannery BP. Numerical Recipes - The Art of Scientific Computing (3rd Edition). 32 Avenue of the Americas, New York NY 10013-2473, USA: Cambridge University Press; 2007.
17. Black MJ, Sapiro G, Marimont DH, Heeger D. Robust anisotropic diffusion. IEEE Transactions on Image Processing. 1998;7(3):421–432.
18. Roger SM, Harston GWJ, Kilburn-Toppin F, Matheson T, Burrows M, Gabbiani F, et al. Spatiotemporal receptive field properties of a looming-sensitive neuron in solitarious and gregarious phases of desert locust. Journal of Neurophysiology. 2010;103:779–792.
19. O'Shea M, Williams JLD. The anatomy and output connections of a locust visual interneurone: the lobula giant movement detector (LGMD) neurone. Journal of Comparative Physiology. 1974;91:257–266.
20. O'Shea M, Rowell CHF. A spike-transmitting electrical synapse between visual interneurons in the locust movement detector system. J Comp Physiol A. 1975;97(2):143–158.
21. Rind FC. A chemical synapse between two motion detecting neurones in the locust brain. Journal of Experimental Biology. 1984;110:143–167.
